# The role of immunotherapy in targeting tumor microenvironment in genitourinary cancers

**DOI:** 10.3389/fimmu.2025.1506278

**Published:** 2025-04-07

**Authors:** Ecem Kalemoglu, Yash Jani, Kubra Canaslan, Mehmet Asim Bilen

**Affiliations:** ^1^ Department of Internal Medicine, Rutgers-Jersey City Medical Center, Jersey City, NJ, United States; ^2^ Department of Basic Oncology, Health Institute of Ege University, Izmir, Türkiye; ^3^ Medical College of Georgia, Augusta, GA, United States; ^4^ Department of Medical Oncology, Dokuz Eylul University, Izmir, Türkiye; ^5^ Department of Hematology and Medical Oncology, Winship Cancer Institute of Emory University, Atlanta, GA, United States; ^6^ Department of Urology, Emory University School of Medicine, Atlanta, GA, United States

**Keywords:** immunotherapy (IO), immune checkpoint inhibitors (ICI), PD-1, PD-L1, T cells, genitourinary cancers (GU), tumor microenvironment (TME)

## Abstract

Genitourinary (GU) cancers, including renal cell carcinoma, prostate cancer, bladder cancer, and testicular cancer, represent a significant health burden and are among the leading causes of cancer-related mortality worldwide. Despite advancements in traditional treatment modalities such as chemotherapy, radiotherapy, and surgery, the complex interplay within the tumor microenvironment (TME) poses substantial hurdles to achieving durable remission and cure. The TME, characterized by its dynamic and multifaceted nature, comprises various cell types, signaling molecules, and the extracellular matrix, all of which are instrumental in cancer progression, metastasis, and therapy resistance. Recent breakthroughs in immunotherapy (IO) have opened a new era in the management of GU cancers, offering renewed hope by leveraging the body’s immune system to combat cancer more selectively and effectively. This approach, distinct from conventional therapies, aims to disrupt cancer’s ability to evade immune detection through mechanisms such as checkpoint inhibition, therapeutic vaccines, and adoptive cell transfer therapies. These strategies highlight the shift towards personalized medicine, emphasizing the importance of understanding the intricate dynamics within the TME for the development of targeted treatments. This article provides an in-depth overview of the current landscape of treatment strategies for GU cancers, with a focus on IO targeting the specific cell types of TME. By exploring the roles of various cell types within the TME and their impact on cancer progression, this review aims to underscore the transformative potential of IO strategies in TME targeting, offering more effective and personalized treatment options for patients with GU cancers, thereby improving outcomes and quality of life.

## Introduction

Cancer is a leading global cause of death (1), with genitourinary (GU) cancers—kidney, prostate, bladder, and testicular—contributing significantly (2). Based on recent statistics from the International Agency for Research on Cancer, kidney, bladder, and prostate cancers are the tenth, sixth, and second most common cancers among men, respectively (2, 3). Chemotherapy, radiotherapy, and surgery have advanced GU cancer treatment (4). However, cancer’s complexity, especially the tumor microenvironment’s (TME) role, challenges long-term remission.

The TME is a complex ecosystem comprising cancer cells, immune cells, stromal cells, and cytokines within a supportive extracellular matrix (ECM). The interactions within the TME play a critical role in cancer pathogenesis, impacting treatment efficacy by evading immune surveillance and promoting tumor growth and metastasis. Understanding these interactions is crucial to effective cancer therapies (5–9).

Immunotherapy (IO) brings new potential to GU cancer treatment, targeting cancer with fewer toxicities than traditional methods. By leveraging the immune system to counter cancer’s immune evasion, IO approaches like immune checkpoint inhibitors (ICI), therapeutic vaccines, and adoptive cell therapies (ACT) target unique TME interactions, promising better outcomes and quality of life (10–12).

This article provides a comprehensive overview of current treatment strategies for GU cancers, focusing on IO that target the TME. We will explore the various cell types within the TME, their significance in cancer progression, and their potential as targets for novel therapeutic interventions.

## Genitourinary cancers

GU cancers affect the urinary and reproductive organs, including the prostate, bladder, kidneys, testicles, and penis. Prostate cancer is the most common, with over 1.4 million cases yearly, followed by bladder and kidney cancers. Treatments vary, including surgery, chemotherapy, IO, and targeted therapies like PARP inhibitors and VEGF inhibitors (13–16).

Targeting immune cells in the TME has shown promise, especially with ICI. Urothelial and kidney carcinomas, known for their high immunogenicity, respond well to ICI, especially in localized and metastatic RCC and urothelial carcinoma. In contrast, testicular germ cell tumors often have an immunosuppressed microenvironment, making them less responsive to ICI. Penile carcinomas, with strong immunogenic characteristics, are potential candidates for IO, though clinical trials are still in the early stages (9, 17–19).

## Tumor microenvironment

The TME is a dynamic landscape crucial for cancer initiation, progression, and metastasis. It includes a heterogeneous mix of non-cancerous cells like fibroblasts, immune cells, endothelial cells, and ECM components. These elements influence cancer cell behavior, growth, and therapy response. The TME’s low pH, hypoxia, and high-pressure impact therapy efficacy. TME interactions can hinder the immune system, drive drug resistance, and support tumor growth and metastasis by secreting growth factors, cytokines, and chemokines (8, 20, 21).

Recent research targets the TME to disrupt its support for cancer cells. Strategies include modulating the immune system to enhance anti-tumor responses, inhibiting angiogenesis, and altering ECM properties to improve drug delivery and efficacy. Uncovering the molecular signals in cancer cell-TME interactions could reveal new therapeutic targets, paving the way for more effective, side-effect-free treatments (7, 9, 22–24).

## Immune cell types in TME

The TME is where immune cells either promote or inhibit cancer. This dynamic landscape is populated by various immune cell types, including CD8^+^ cytotoxic T-lymphocytes (CD8^+^) and CD4^+^ helper T-cells (CD4^+^), which fight tumor cells, and regulatory elements like regulatory T-cells (Tregs) and myeloid-derived suppressor cells (MDSC) that help tumors evade immune detection. Tumor-associated macrophages (TAMs) and dendritic cells (DC) further complicate this interplay, with their actions ranging from tumor suppression to support (25–27). Cancer cells within the TME can avoid detection and destruction by the host immune system through proximity to healthy cells or communication via cytokines. The balance of these forces within the TME significantly impacts the effectiveness of cancer therapies, particularly IO (9, 28).

### T cells in TME

T cells in the TME are essential to the body’s defense, identifying and eliminating pathogens and cancer cells as part of the adaptive immune system (29). Maturing in the thymus and expressing T-cell receptors (TCRs), T-cells are categorized by their CD4 or CD8 glycoproteins (30, 31).

Naive CD4+T-cells, upon encountering major histocompatibility complex (MHC)-II on APCs like DCs and macrophages, get activated and differentiate into specific subtypes, guided by the cytokine environment in secondary lymphatic tissues such as lymph nodes (LN) (32). These subtypes— T-helper (Th)1, Th2, Th9, Th17, Th22, Tregs, and follicular helper T-cells (Tfh)—each have distinct cytokine profiles and roles in immunity, ranging from anti-tumorigenic to immunosuppressive. Th1 cells release anti-tumor cytokines like interleukin (IL)-2, interferon (IFN)-γ, and tumor necrosis factor (TNF)-α, while Th2 cells release immunosuppressive IL-4, IL-5, IL-10, and IL-13. Th9 produces IL-9, Th17 releases IL-17, Th22 produces IL-22, Tregs primarily secrete immunosuppressive IL-10 and transforming growth factor (TGF)-β, and Tfh cells release IL-4 and IL-21 (29, 31).

While Th cells do not directly eliminate cancer cells, they activate key immune players, including CD8+T-cells, which target malignant cells, B-cells that produce antibodies, and macrophages that consume pathogens (32, 33). In the TME, Th1, and Th9 subsets of CD4+T-cells enhance CD8+T-cell antitumor activity through cytokines like IL-2, IFN-γ, and IL-9, correlating with improved outcomes across cancers (34, 35). Tregs, another subset of CD4+ T-cells, mainly prevent autoimmunity but, in the TME, promote cancer progression by suppressing effector T-cells and fostering an immunosuppressive environment. They aid tumor growth and may facilitate metastasis with a complex cytokine profile (36, 37).

CD8+T-cells are key in cancer defense, maturing into cytotoxic cells through TCR engagement with MHC-II on APCs, supported by co-stimulatory signals (CD28/CD80/CD86) and cytokines like IL-2, IFN-γ, and IL-9 from Th1 and Th9 cells (26, 33, 35). Besides directly killing cancer cells, they inhibit tumor growth by blocking angiogenesis via IFN-γ (38).

Natural killer T (NKT) cells, a unique subset of CD1d-restricted T-cells, also bridge innate and adaptive immunity by expressing both TCR and natural killer (NK) cell receptors. NKT-cells respond rapidly to glycolipids and stress proteins and play diverse roles in immune functions, including tumor surveillance, self-tolerance, and regulation of autoimmune diseases. They are critical in early tumor responses, with Th1-like NKT-cells activating tumor-specific T and NK cells (39, 40).

### Tumor-associated macrophages in TME

TAMs are key immune cells infiltrating the TME, impacting tumor angiogenesis, metastasis, and prognosis in solid tumors (41). They are categorized into M1-macrophages, with anti-tumor properties, and M2-macrophages, which support tumor growth. M1-type macrophages kill tumor cells by releasing reactive oxygen species (ROS), nitric oxide (NO), and pro-inflammatory cytokines, TNF-α, IL-6, IFN-γ, over days and through antibody-dependent cell-mediated cytotoxicity (ADCC) within hours (41–43). In contrast, M2-type macrophages promote tumor progression by secreting anti-inflammatory cytokines, IL-1, IL-4, IL-10, and pro-angiogenic factors, vascular-endothelial-growth-factor (VEGF), IL-8, recruiting Tregs and MDSCs, and releasing matrix metalloproteinases (MMPs) to remodel the ECM, aiding metastasis (9, 41, 42, 44, 45).

### Dendritic cells in TME

DCs, the primary APCs, bridge innate and adaptive immunity (46). They are mainly divided into plasmacytoid (pDC) and myeloid dendritic cells (mDCs). pDCs, known for type-I IFN production, have limited antigen-presentation abilities, with a debated role in the TME; while some suggest they promote immunosuppression, their release of IFN-γ and TNF-α indicates potential for IO (9, 47). Conventional DCs (cDC), a subset of mDCs, include cDC1, which cross-present antigens to CD8+ T-cells on MHC-I to drive antitumor responses, and cDC2, which primarily activate CD4+ T-cells through MHC-II (48).

Immature DCs excel in antigen capture but have low co-stimulatory and cytokine levels. Upon exposure to pathogens or damaged tissue, they mature, reduce antigen uptake, upregulate MHC-II and CCR7, and migrate to LN, driving T-cell responses through TNF-α, IL-12, IL-6, and IL-8 secretion, improving cancer treatment outcomes (49, 50).

### Natural killer cells in TME

NK cells, essential cells in innate immunity against cancer, eliminate tumor cells directly without prior sensitization, guided by a balance of activating and inhibitory receptors that detect abnormalities and release cytotoxic granules (51). They are also crucial for immune surveillance, preventing tumor establishment by eradicating malignant cells and secreting pro-inflammatory cytokines like IFN-γ, which amplify the antitumor response of other cells (51–53). NK cells are classified into CD56^bright^CD16^-^ and CD56^dim^CD16^+^ subpopulations. CD56^bright^ NK cells respond to pro-inflammatory cytokines, influencing adaptive immunity, while CD56^dim^ NK cells drive direct cytotoxicity against infected and cancerous cells (54, 55). NK can also eliminate MHC-I deficient tumor cells, linking the innate and adaptive immune systems (54, 55).

### Myeloid-derived suppressor cells in TME

Myeloid cells, comprising both mature cells like neutrophils and macrophages and immature monocytes and precursors, play key roles in cancer immunity. Cancer disrupts normal myeloid differentiation, leading to an increase in abnormally activated MDSC, which suppresses immune functions. MDSCs include polymorphonuclear (PMN-MDSC), making up over 80% of MDSCs in cancer tissue, and mononuclear (M-MDSC) subtype. MDSCs expansion is mostly controlled by tumor cells and immune cells via chemokines, TLRs, and IFN- γ pathways (56). Elevated PMN-MDSC levels are linked to poorer immune responses in many solid tumors (57–59).

### Neutrophils in TME

Neutrophils, the most common innate immune cells in the body, often expand in solid tumors, generally correlating with poor prognosis (60, 61); the impact of neutrophils on solid tumor metastasis is still widely debated since they can exhibit both pro- and anti-metastatic roles (62). They promote cancer growth by producing MMP-9, aiding angiogenesis (60, 63), and forming neutrophil extracellular traps (NETs), which support tumor growth and metastasis in many cancer types (60, 63). On the other hand, studies suggest neutrophils have an anti-metastatic effect through H2O2 cytotoxicity, regulated by C-C chemokine ligand (CCL)-2 (60, 62). Given their impact on treatment outcomes, targeting neutrophils’ tumor-promoting functions may improve and enhance anti-cancer treatment effectiveness (60).

### B-cells in TME

B-cells, typically part of adaptive immunity, have a complex role in the TME, influencing cancer progression and therapy response (64). In TME, B-cells predominantly reside in tertiary lymphoid structures (TLS), where they mature and inhibit tumor growth, presenting antigens to T-cells and supporting anti-tumor responses. Plasma cells derived from B-cells produce antibodies that can enhance the anti-tumor activities of macrophages and NK. Conversely, B-cells in underdeveloped TLS can promote tumor-supportive inflammation and may transform into regulatory B-cells (Bregs).

### Other cell types in TME

Cancers form intricate ecosystems consisting of tumor cells, various non-cancerous cells, and a modified ECM. In addition to immune cells, cancer-associated fibroblasts (CAFs), endothelial cells, pericytes, adipocytes, tumor stem cells (TSC), and other resident tissue cells (7, 65).

### Tertiary lymphoid structures in TME

Traditionally, adaptive immune responses against cancer develop in secondary lymphoid organs (SLOs), where DCs present tumor antigens to T-cells, initiating B-cell activation and germinal center (GC) formation. This results in the proliferation of effector and memory T and B cells that target cancer cells. However, research shows that antitumor responses also occur within tumors in structures called TLS, which are ectopic lymphoid aggregates. Similar to SLOs, TLS is composed of an organized follicular B cell zone with GC, T-cell zones, and antigen-presenting DCs and plays a critical role in local immune defenses, leading to the activation of immune cells and the production of memory cells (66).

TLS formation in tumors can arise from chronic inflammation without traditional lymphoid tissue inducer (LTi) cells. Immune-stromal interactions release chemokines (CXCL13, CXCL12, CCL21, CCL19) and adhesion molecules, recruiting lymphocytes via high endothelial venules (HEVs) to establish distinct T- and B-cell zones (67). TLS presence correlates with higher CD8+ and CD4+ T-cell densities in the TME and improved prognosis in many solid cancers. Mature TLS, particularly those with GC, enhances antitumor immunity by generating memory B-cells and plasma cells that secrete high-affinity antibodies. Studies show B-cells enriched in responders to ICI localize within TLS, making B-cell-rich TLS a stronger predictor of ICI response and survival than T-cells alone (66, 68, 69). Additionally, Treg depletion activates CD8+ T-cells, promoting HEV development and TLS formation, enhancing T-cell infiltration and tumor destruction. This suggests potential targets for IO (70).

**Figure 1:**
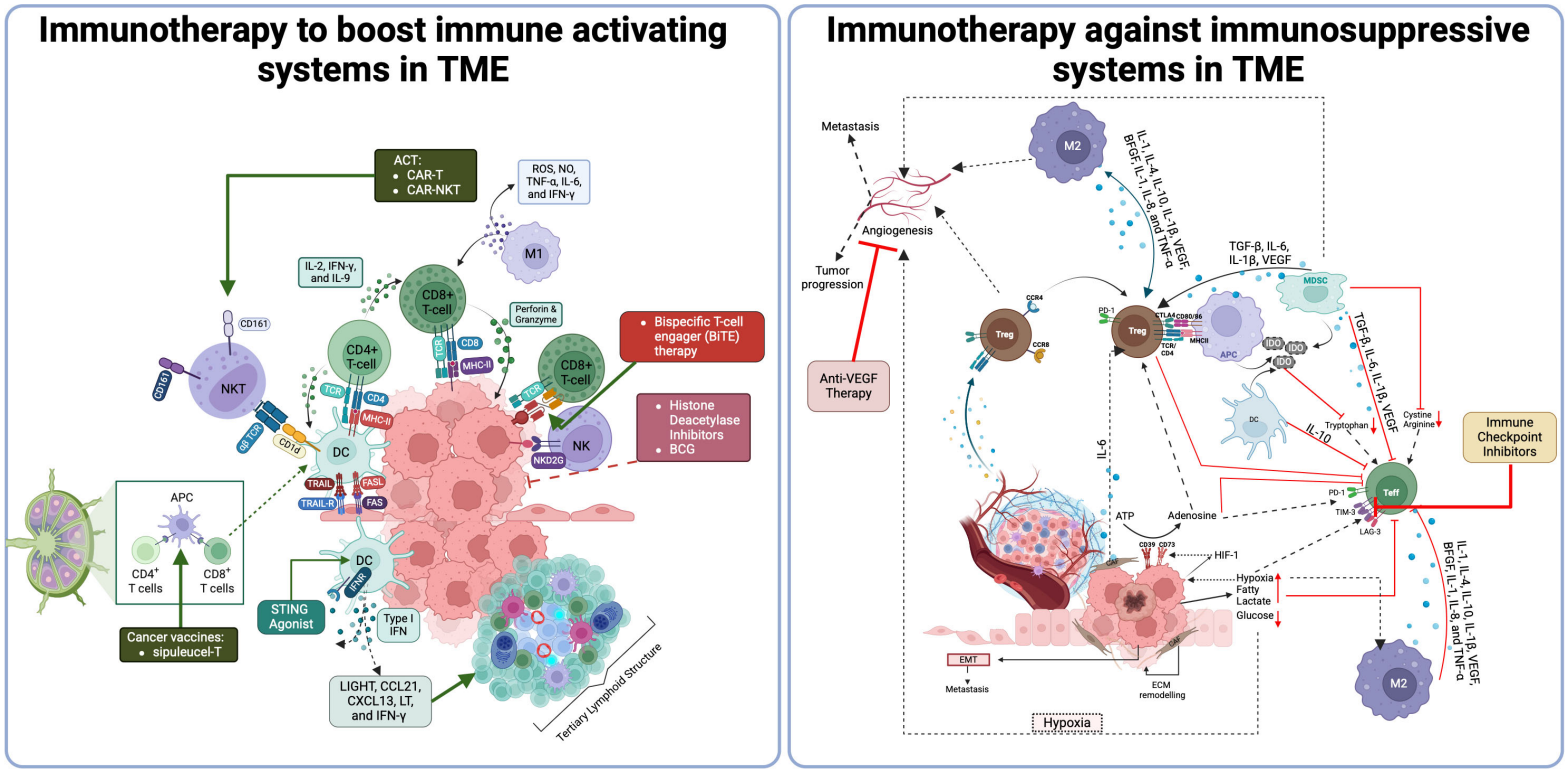
Tumor microenvironment and Its components (411). Th, T helper; Treg, Regulatory T-cell; Tfh, Follicular T-cell; Breg, Regulatory B-cell; DC, dendritic cell; pDC, Plasmacytoid DC; cDC, Conventional DC; NK, Natural killer cell; MDSCs, Myeloid-derived suppressor cells; M-MDSCS, Monocytic-MDSCs; PMD-MDSCs, Granulocytic-MDSCs; CXCL13, C-X-C Motif Chemokine Ligand 13; CXCL12, C-X-C Motif Chemokine Ligand 12; CCL21, C-C Motif Chemokine Ligand 21; CCL19, C-C Motif Chemokine Ligand 19; LTαβ, Lymphotoxin Alpha-Beta; LTBR, Lymphotoxin Beta Receptor.

TME and its components are schematized in **Figure 1**.

## Immunotherapy approaches

IO boosts the body’s cancer defenses, but response rates vary, and mechanisms are not fully understood. Targeting TME refines IO and reveals new therapies. While T-cells are well-studied, other immune cells like DCs, macrophages, NK cells, and B-cells also influence cancer and therapy responses, showing TME complexity. Integrating IO with chemotherapy or targeted therapies in settings like neo-adjuvant, adjuvant, and metastatic is actively investigated. Many IOs, such as ICI, oncolytic virus therapies, cancer vaccines, cytokine therapies, and ACT, have been developed for cancer treatment (71, 72).

ICI, key in IO, enhances antitumor immunity by blocking checkpoints like CTLA-4, PD-1, and PD-L1, allowing T-cells to target cancer cells. CTLA-4 binds B7 antigens on APCs, inhibiting T-cell activation; blocking CTLA-4 boosts cytotoxic T-cell activity, leading to Ipilimumab’s development, the first ICI (73–75). PD-1 regulates immunity by binding to PD-L1/PD-L2 on tumor and myeloid cells, suppressing T-cell activation, especially under inflammatory stimuli like INF-γ, IL-1α, or IL-27 (75–77) and hypoxia by hypoxia-inducible factor-1α (HIF-1α) (78–80). ICI like Nivolumab, Pembrolizumab (anti-PD-1), Atezolizumab, Avelumab (anti-PD-L1), and Ipilimumab (anti-CTLA-4) block these inhibitory checkpoints and enable T-cells to attack cancer cells (9, 81, 82).

Though primarily on T-cells, PD-1 is also found on myeloid cells like macrophages, MDSCs, B-cells, DCs, and NKs in the TME. PD-1 expression on myeloid cells boosts immunosuppressive abilities, promotes the M2 macrophage phenotype, and increases IL-6 production, underscoring ICI’s broad influence on TME dynamics (83, 84).

Since anti-PD-1/PD-L1 molecules are widely used in different cancer types, this strategy may result in the upregulation of alternative checkpoint targets (85). LAG-3 is expressed on different immune cells and has an inhibitory role on cytotoxic T-cell functions as well as stimulation on Tregs (86). In RCC, LAG-3 overexpression is found to be associated with CD8+ T-cell exhaustion and anti-PD-1 resistance (87). TIM-3 can be found in immune cells and non-immune cells, such as tumor-associated endothelial cells. Interaction between TIM-3 and its ligands results in CD8+ T-cell exhaustion (88). TIM-3 and PD-1 co-expression on T-cells leads to poor prognosis in clear cell renal cell carcinoma (ccRCC) (89). T-cell immunoreceptor with immunoglobulin and immunoreceptor tyrosine-based inhibition motif domain (TIGIT) is another checkpoint molecule expressed exclusively on T-cells and NK cells and suppresses the cytotoxic activities of NK cells (90). In muscle-invasive bladder cancer, high infiltration of intratumoral TIGIT+ T-cells results in immunosuppressive TME (91). Also, targeting TIGIT leads to loss of the ability of bladder cancer cells to metastasize (92).

The association between the presence of TLS and clinical benefits in cancer patients highlights their potential as prognostic and predictive factors despite some studies suggesting a possible negative impact. Strategies to trigger TLS neogenesis in both low and high-immune activity tumors, especially when combined with ICI, show promise for advancing cancer treatment. However, leveraging TLSs to enhance immune activation to boost antitumor immunity remains challenging. Multiple approaches have been developed to enhance local antitumor immunity to induce TLS formation, including manipulating LN properties to improve IO efficacy with cancer vaccines, ICI therapy, and ACT (66, 67). Additionally, research focuses on TLS-inducing chemokines and cytokines to create more effective IO. Agents like LIGHT, CCL19/CCL21-CCR7, CXCL13-CXCR5, LT, and IFN-γ are key to lymphogenesis and TLS formation and may be used to develop artificial lymphoid tissues within the TME. For example, LIGHT-VTP targets tumor vessels and may induce HEV and TLS formation through a self-amplifying loop. The combination of LIGHT-VTP with ICI can promote the induction of memory T-cells and effector T-cells in the TME, improving prognosis. Incorporating anti-VEGF therapy with this regimen could enhance HEVs and the accumulation of T-cells in TME, further positively impacting prognosis (67, 93). Furthermore, some *in-vitro* studies showed that biomaterials can be used as a delivery strategy. These biomaterials serve as immune niches to deliver lymphogenesis-inducing chemokines/cytokines and cells and initiate intratumoral immune sensitization through artificial LN, thus boosting antitumor immunity. Biomaterial-based IO holds the potential to advance the development of future cancer treatments (67).

Stimulator of interferon genes (STING), a cytosolic protein that detects DNA, activates upon binding cGAMP, which then stimulates the expression of inflammatory genes such as IFN. Tumors often show genetic instability and high cytosolic DNA, inherently activating STING and promoting inflammation. However, tumors can also disrupt STING pathways to evade immune detection. Yet, dying tumor cells can still trigger STING activation in the tumor environment, potentially enhancing therapy with synthetic STING agonists (94). Additionally, STING activation with agonists enhances the production of chemokines and cytokines that induce TLS within TME and promote DC maturation. This leads to increased proinflammatory immune infiltration and the development of non-classical TLS, contributing to the prevention of tumor growth (95). Also, STING agonists, combined with treatments like radiotherapy, chemotherapy, vaccines, monoclonal antibodies, or ICIs, show strong potential for enhancing therapeutic synergy (96).

ACT, a novel IO approach, involves modifying and reinfusing a patient’s T-cells, using methods such as TCR-engineered T-cells (TCR-T), tumor-infiltrating lymphocytes (TIL) transfer therapy and chimeric antigen receptors (CAR)-T cells, especially in advanced cancers. TCR-T cells target tumor cells based on antigens presented by Human-Leukocyte-Antigen (HLA)-I, while CAR-T cells are tailored ex-vivo to specifically attack surface antigens, using an antibody-derived scFv. This prevents tumor cells from evading the immune system by downregulating HLA-I. Once reintroduced, these CAR-T cells initiate an immune response that eradicates tumor cells expressing the target antigen. This approach has shown encouraging outcomes in treating solid tumors; however, this therapy has shown a better clinical response in hematological malignancies (97–99). CAR-NKT-cells are also being studied as a more accessible alternative to autologous CAR-T cells, which are costly and time-intensive to produce, with limited accessibility for patients lacking adequate T-cells. NKT-cells, a rare type of αβ T-cell, express an invariant TCR-α chain and NK markers, allowing them to target tumors without triggering graft-versus-host disease. These cells recognize the CD1d molecule and exhibit potent tumor-killing capabilities, tumor infiltration, and the ability to bridge innate and adaptive immunity, making them promising candidates for new cancer treatment (100).

Tumor-derived chemokines often inhibit DC infiltration and antigen uptake (47). Recent findings highlight the importance of cDC1 in the TME, particularly in ICI responses and as targets for enhancing T-cell proliferation and antitumor activity. Novel approaches, such as CD40-ligation and protein kinase-C agonists, aim to improve cDC function, boosting antigen cross-presentation and protective immunity in solid tumors (101). Additionally, blocking Tim-3 was shown to enhance the anti-tumor immunity of STING agonists by unleashing CD4+ T-cells through the regulation of cDC2 (102). Combining DC therapy with other IO approaches to overcome the immunosuppressive TME represents a promising future direction for cancer treatment, highlighting DCs’ evolving role in IO.

Emerging bispecific antibodies (BsAbs) enhance tumor immuno-oncology by recruiting T-cells to tumor cells. BsAbs are classified into three types: targeting two tumor antigens, a tumor antigen, and an immune molecule, or two immune molecules. Bispecific T cell engagers (BiTEs) fall into the second group (103), independently activating T-cells to drive cytotoxicity, cytokine release, and B-cell activation by targeting both a tumor antigen and CD3e, leading to T-cell-dependent tumor cell destruction (103–105). BsAbs in urological cancers target a range of antigens to enhance immune response and tumor destruction. PSMA × CD3 is widely studied in metastatic castration-resistant prostate cancer (mCRPC). Other prostate cancer targets include PSMA × CD28 and KLK2 × CD3. In renal cell carcinoma (RCC), BsAbs target ENPP3 × CD3, HER2 × HER3, and PD-1 × CTLA-4. Bladder cancer therapies include CTLA-4 × OX40 and CD3 × B7-H3 BATs. Additionally, CD155 Bi-armed T cells, CD3 × STEAP-1, and EpCAM-targeting catumaxomab show promise in various urological malignancies. These diverse targets aim to enhance immune activation, reduce tumor growth, and improve clinical outcomes (106).

Targeting tumor-specific antigens and developing tumor vaccines are essential for improved treatments. In 2010, the FDA approved PROVENGE (sipuleucel-T), the first therapeutic cancer vaccine for advanced hormone-refractory prostate cancer. Since the start of IO, people have invested numerous passion and efforts in researching tumor vaccines. There are mainly three types of cancer vaccines: cell, peptide, and nucleic acid vaccines, which aim to increase TIL infiltration or enhance their antitumor activity. Despite the promise, their clinical application remains limited (72).

Histone deacetylases (HDACs) and acetyltransferases, crucial for regulating chromatin structure and gene expression, become therapeutic targets through HDAC inhibitors (HDACis) like entinostat, panobinostat, and chidamide. These inhibitors, studied in combination with ICI and TKIs, not only modify chromatin structure to potentially enhance tumor antigen presentation and T-cell activation but also reverse gene silencing. This promotes tumor suppressor activity and boosts immune responses by upregulating MHC I and II, significantly improving the efficacy of IO (107–109). In addition, Entinostat, a class-I HDAC inhibitor, increases PD-L1 expression, enhances NK cell and CD8+ T-cell activity in tumors, and improves neoantigen-specific immune responses and suppresses Tregs and MDSC, which bolsters its effectiveness in combination with other IO (110).

Like histone acetylation and deacetylation, DNA methylation, governed by DNA methyltransferases (DNMTs), is key for regulating gene expression and silencing tumor suppressor genes in cancer. DNMT inhibitors (DNMTis) like azacitidine and decitabine reactivate these genes and enhance tumor antigen presentation by boosting the expression of tumor-associated antigens (TAA) and cytokines such as IL-2 and IFN-γ. They increase the efficacy of cytotoxic T lymphocytes and NK cells, inhibit Tregs, redirect MDSC towards DC phenotypes, and promote an anti-tumor M1 phenotype in macrophages. Therefore, combining DNMTis with chemotherapy or ICI could offer valuable strategies for developing effective cancer treatments (111, 112).

Ionizing radiation therapy (RT) enhances T-cell responses, particularly boosting the function of CD8+ T-cells, making it a valuable adjunct to PD-1/PD-L1 inhibitors in cancer treatment. RT upregulates tumor antigen presentation and fosters CD8+ T-cell infiltration, which is crucial for effective anti-tumor immunity. Additionally, RT induces radiation induced cell death, a process that promotes the release of tumor antigens and damage-associated molecular patterns. These, in turn, activate DC and amplify the anti-tumor T-cell response. However, RT alone may not completely eradicate tumors, partly because of immune-suppressive pathways such as the PD-1/PD-L1 axis. Combining RT with ICI addresses these limitations, significantly enhancing CD8+ T-cell activity, which leads to improved systemic immune responses and potential tumor regression (113).

**Figure 2:**
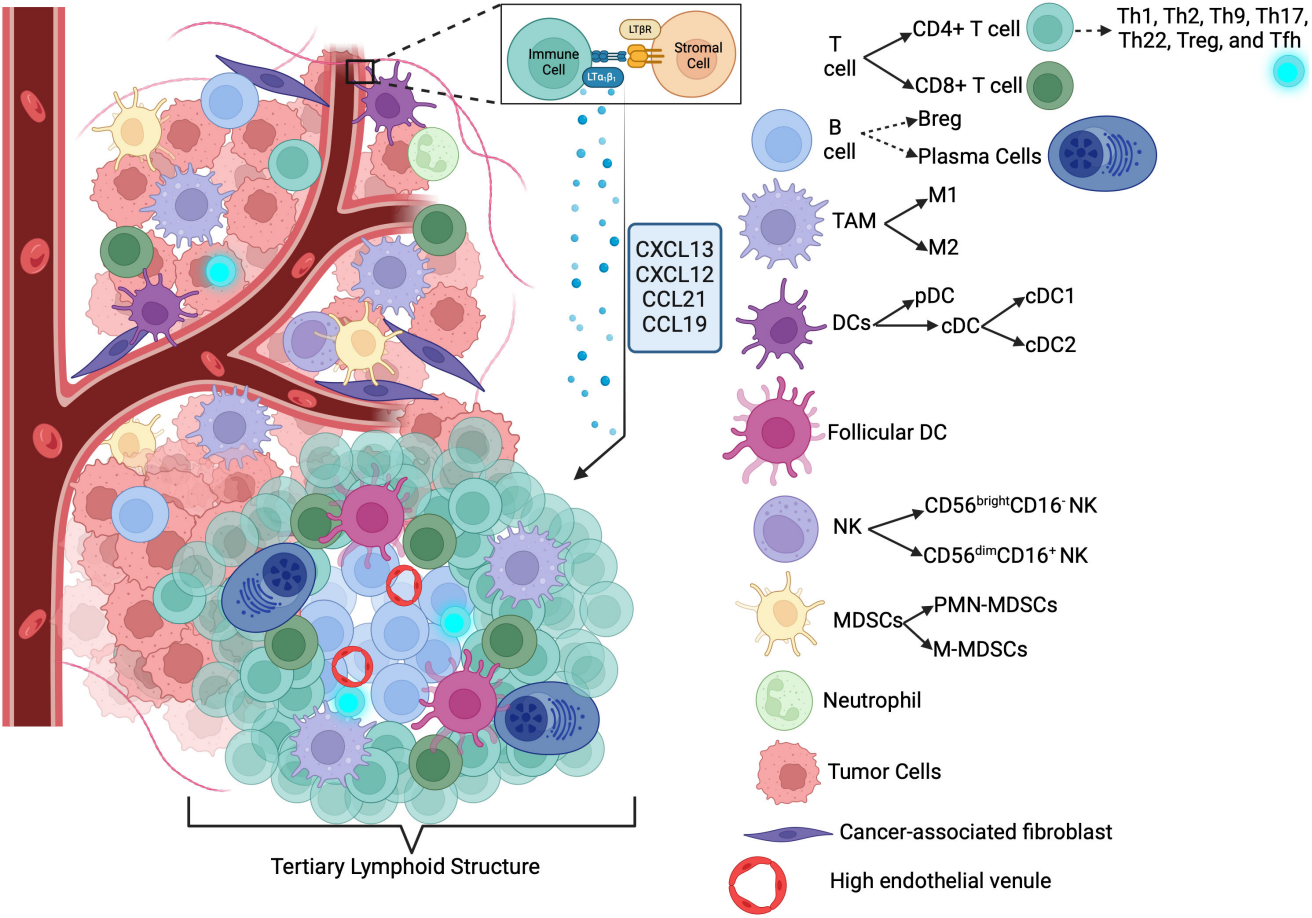
Immune activating/suppressive networks in TME and different immunotherapy strategies (412). ACT, Adoptive Cell Transfer; CAR-T, Chimeric Antigen Receptor T-cell; CAR-NK, Chimeric Antigen Receptor Natural Killer cell; IL-2, Interleukin-2; IFNγ, Interferon gamma; IL-9, Interleukin-9; MHC I, Major Histocompatibility Complex Class I; NKT, Natural Killer T cell; CD8+ T-cell, CD8 Positive T-cell; M1, Type 1 Macrophage; DC, Dendritic Cell; BiTE, Bispecific T-cell Engager; HDAC, Histone Deacetylase; BCG, Bacillus Calmette-Guérin; STING, Stimulator of Interferon Genes; LIGHT, A type of cytokine; CCL21, Chemokine (C-C motif) ligand 21; CXCL13, Chemokine (C-X-C motif) ligand 13; LT, Lymphotoxin; IFNγ, Interferon gamma; TME, Tumor Microenvironment; ROS, Reactive Oxygen Species; NO, Nitric Oxide; TNFα, Tumor Necrosis Factor alpha; IL-6, Interleukin-6; VEGF, Vascular Endothelial Growth Factor; M2, Type 2 Macrophage; TGF-β, Transforming Growth Factor beta; IL-1β, Interleukin-1 beta; MDSC, Myeloid-derived Suppressor Cell; IDO, Indoleamine 2,3-dioxygenase; PD-1, Programmed Death-1; Treg, Regulatory T cell; HE-1, Hypoxia-inducible factor 1; ATP, Adenosine Triphosphate; ECM, Extracellular Matrix; PD-L1, Programmed Death-Ligand 1.

Immune cells, immunosuppressive networks in TME, and different IO strategies in TME are summarized in **Figure 2**.

## Predictive biomarkers for immunotherapy response

ICI has shown strong clinical success, but not all patients benefit, making predictive biomarkers essential for personalized treatment. PD-L1, the first biomarker for anti-PD-1 therapy, has limitations due to its regulation by immune pathways. Resistance to ICI is linked to mutations in interferon and antigen presentation pathways, while T-cell inflammatory gene expression and somatic copy number variation are associated with better outcomes (114, 115).

Current IO biomarkers fall into three main categories: surface markers like PD-L1, detected via immunohistochemistry, genetic biomarkers such as tumor mutation burden (TMB), mismatch repair-deficient (dMMR), high microsatellite instability (MSI), neoantigens, and antigen presentation mutations, requiring genomic analysis and circulating tumor DNA (ctDNA), analyzed from blood samples. Some have been validated in phase-III trials and are clinically used while research continues to identify new biomarkers (114).

dMMR tumors arise from mutations in MMR genes (MLH1, PMS2, MSH2, MSH6), leading to errors in DNA replication, particularly in microsatellites, resulting in high MSI (MSI-H). Found in 2-4% of cancers, MSI-H/dMMR tumors have increased somatic mutations, higher neoantigen loads, and elevated immune checkpoint protein expression. These tumors often respond well to ICI, with studies confirming the efficacy of PD-1/PD-L1 blockade. Pembrolizumab has been shown to have strong antitumor activity in MSI-H/dMMR all solid tumors after prior treatment failure (116).

TMB quantifies the number of mutations per megabase in tumor cells and is a key biomarker for predicting IO response. Tumors with high TMB generate more immunogenic neoantigens, enhancing T-cell recognition and response to anti-PD-1 therapies. TMB quantifies tumor mutations and is typically assessed via whole-exome sequencing (~30 Mb) or panel-based assays like FoundationOne CDx (~0.8 Mb). The FDA approved pembrolizumab for tumors with TMB ≥10 muts/Mb based on the KEYNOTE-158 study, though questions remain about applying a universal TMB threshold across cancers. Factors like biopsy site, tumor heterogeneity, and sequencing depth affect TMB reliability. Blood-based TMB, measured via cell-free DNA, offers a non-invasive alternative but requires further validation (117, 118).

Neoantigens are tumor-specific proteins resulting from nonsynonymous mutations, making them recognizable targets for T cells. They are absent in normal tissues and must have corresponding TCRs for immune recognition (119). As potential biomarkers for ICI, neoantigens may better reflect tumor immunogenicity than MSI, MMR, or TMB. High-affinity binding to MHC increases the likelihood of an immune response, but validating their immunogenic quality remains a challenge, and currently, neoantigens mainly support other biomarkers (114).

ctDNA is tumor-derived DNA fragments from apoptotic and necrotic cancer cells in the bloodstream, serving as a non-invasive biomarker for cancer detection and monitoring (120). With a short half-life (16 min–2.5 hrs), ctDNA enables real-time tracking and early recurrence detection (121). It is detected via digital PCR, next-generation sequencing (NGS), and methylation profiling.

GU cancers, ctDNA has emerged as a powerful liquid biopsy tool for early detection, prognosis, therapy response assessment, and resistance monitoring (122). In mCRPC, the detection of androgen receptor (AR) mutations, amplifications, and the AR-V7 splice variant via ctDNA has been shown to predict resistance to AR pathway inhibitors, and these changes can be found in liquid biopsy months before actual disease progression (123, 124). Additionally, BRCA1/2 and ATM mutations, PTEN loss, or MSI may be detected by ctDNA and guide the treatment decision for prostate cancer patients (125, 126). Moreover, ctDNA changes correlate with survival, track AR mutations, and detect resistance, guiding treatment adjustments in prostate cancer (127, 128).

In bladder cancer, urinary ctDNA, particularly sensitive in detecting TERT promoter mutations, correlates with tumor burden and recurrence risk (129). Pivotal studies have shown ctDNA’s significant prognostic value in managing muscle-invasive bladder cancer (MIBC) (130, 131). The ABACUS trial demonstrated that ctDNA clearance after neoadjuvant atezolizumab in MIBC patients predicts pathological complete responses and relapse-free outcomes (132). The IMvigor010 trial underscored ctDNA’s role in stratifying patients for adjuvant atezolizumab, showing significant benefits in disease-free survival and overall survival for ctDNA-positive patients (133). Lastly, the VOLGA study found that ctDNA-negative status or clearance after neoadjuvant treatment with durvalumab, tremelimumab, and enfortumab vedotin in cisplatin-ineligible MIBC patients was associated with improved event-free survival and reduced likelihood of being upstaged at surgery (134).

Despite its potential, renal cell carcinoma (RCC) presents unique challenges for ctDNA detection due to low levels of ctDNA (135). However, higher levels of tumor methylation in ctDNA have been associated with advanced disease, indicating its potential utility in assessing tumor progression and guiding therapeutic decisions (136).

The TCR, a highly variable receptor, binds antigens via MHC, activating adaptive immunity against infections and tumors. TCR clonality, which refers to the diversity and expansion of T-cell clones, can be detected using NGS and serves as a key biomarker in IO. TCR clonality, which refers to the diversity and expansion of T-cell clones, can be detected using NGS and serves as a key biomarker in IO (137, 138).

In certain cancers, a few dominant T-cell clones expand significantly in response to TAA, leading to high TCR clonality. This phenomenon is often observed in tumors responding to IO (139). A high degree of clonality suggests an ongoing, tumor-specific immune response, which is generally associated with better treatment outcomes (140). Some tumors, such as prostate cancer, are considered “cold” due to low TCR clonality, making them less responsive to ICI (141).

A highly clonal T-cell response initially indicates tumor recognition, but over time, T-cell exhaustion and immune evasion can occur, allowing the tumor to escape immune control. Tracking TCR clonality over time provides critical insights into whether T-cells remain functional or become ineffective, helping guide treatment adjustments (142, 143). TCR clonality is a powerful immune biomarker for predicting cancer progression, response to IO, and immune resistance mechanisms (144). By integrating TCR sequencing with ctDNA analysis, personalized IO strategies can be developed.

Understanding the spatial organization and interactions of immune cells within the TME is essential for elucidating immune evasion mechanisms and developing effective immunotherapies. Advanced multiplex immunohistochemistry (mIHC)/immunofluorescence (IF) platforms, such as CO-Detection by Indexing (CODEX) and Multiplexed Ion Beam Imaging (MIBI), have revolutionized this field by enabling simultaneous visualization of multiple biomarkers at single-cell resolution (145). CODEX enables highly multiplexed immune cell profiling (146), while MIBI, using metal-tagged antibodies and mass spectrometry, maps immune interactions in the TME (147). These technologies reveal spatial immune dynamics and predictive biomarkers for ICI therapy.

Proximity of CD8+ T cells to PD-L1+ tumor cells can help to predict anti-PD-1 response (148). mIHC/IF-based spatial proteomics identifies tumor-immune niches influencing therapy. Integrating transcriptomics with high-dimensional imaging enhances understanding of immune-tumor crosstalk, underscoring the need for spatial profiling in clinical practice.

TILs are emerging as prognostic and therapeutic biomarkers in ICI therapy, with their phenotype, function, and location influencing outcomes. Many active immune responses form in peritumoral TLS, which act as local immune hubs. Studies link cancer-associated TLS with improved survival and ICI response, suggesting TLS density as a potential biomarker independent of PD-L1 expression. Advanced technologies like mIHC/IF and gene expression profiling help characterize TILs. mIHC/IF, which maps immune markers spatially, has outperformed PD-L1 mIHC, and TMB in predicting ICI response. Digital immune and prognostic scores integrating multiple immune features are being developed to enhance personalized IO strategies (149).

mIHC, identify TLS-associated gene signatures. The 12-chemokine signature (CCL2, CCL3, CCL4, CCL5, CCL8, CCL18, CCL19, CCL21, CXCL9, CXCL10, CXCL11, CXCL13) is widely used to quantify TLSs in solid tumors. High TLS signature scores correlate with better survival and stronger immune responses (150). Its predictive value was validated for immunotherapy response by using publicly available datasets (150, 151), while another study used machine learning and the 12-chemokine signature to identify TLS clusters in ccRCC, revealing differences in survival, immune distribution, and IO response (150, 152).

Some cytokines and chemokines that induce TLS formation also predict ICI response. For instance, chemokine CXCL13 drives TLS formation by recruiting B cells and is linked to better ICI responses in some solid tumors. High CXCL13 expression correlates with prolonged survival and increased CD8+ T-cell infiltration (153–155). A Pan-cancer study showed that T-cells’ expanded signature, including CXCL13 and other genes, are necessary for clinical response to ICI (156). Additionally, CXCL13 expression plus TLS formation was found to predict a favorable response to anti-PD-1 blockade in metastatic urothelial cancer (157). Similarly, studies showed that CXCL13 expression plus ARID1A mutation work together as a combination biomarker to predict a favorable response to ICI in metastatic urothelial cancer (158).

Despite high initial response rates, many patients relapse after CAR-T cell therapy, highlighting the need for predictive biomarkers. While none currently guide patient selection, TAA expression is a key factor, as CAR-T cells rely on TAA presence for efficacy. Tumor recurrence often results from antigen escape, where TAA downregulation—not complete loss—can impair CAR-T function, making TAA density a potential biomarker. Additionally, product quality impacts outcomes, as prior treatments alter T-cell composition in autologous CAR-T therapy. Studies link polyfunctionality, such as cytokine production and memory-like T-cell populations in infusion, to better responses, suggesting these factors could serve as predictive biomarkers with further validation (114).

### Immune cells, TME-driven resistance and strategies to overcome

One of the hallmarks of cancer is avoiding immune destruction, and it is mostly possible with immunosuppressive TME (159). While cytotoxic T-cells drive anti-tumor activity, Tregs, MDSCs, and TAMs play a suppressive role in blunting the cytotoxic T-cell activity and promoting tumor progression (160).

Treg recruitment depends on chemokine receptors like CCR4 and CCR8. Inhibition of CCR4 has demonstrated clinical activity by depleting Tregs in solid tumors in humans when combined with anti-PD1 therapy (161, 162). The co-stimulatory molecule OX40 controls Treg activity and indirectly up-regulates CD4+ T-cells (163). Moreover, CD25 is a prominent surface marker for Tregs, and targeting it with antibodies or cytotoxic agents can enhance antitumor immunity but may risk impairing IL-2-driven T-cell activation. A safer approach is intratumoral injection of CD25-targeting agents, reducing local Treg suppression while preserving overall T cell function (164). Also, NKT-cells can become overstimulated and anergic during tumor progression, leading to cell death or a shift toward immunosuppressive Th2/Tregs states, facilitating tumor growth, and strategies to expand Th1-like NKT-cells may improve antitumor immunity (39, 40).

MDSCs inhibit T-cell activity by depleting essential amino acids like L-arginine and cysteine and producing ROS, damaging cells’ DNA and promoting energy via PD-1/PD-L1 interactions and adenosine (57, 165, 166). It was shown that patients with bladder cancer exhibit higher levels of MDSCs in peripheral blood compared to healthy donors (167). Also, prostate cancer cells secrete a high number of chemokines to attract MDSCs and Tregs (168). Additionally, MDSCs impair NK function by reducing tryptophan through indoleamine 2,3-dioxygenase (IDO) expression, promoting Treg differentiation, and reducing NK cytotoxicity via TGF-ß and IFN-γ (57, 169, 170). The induction of IDO by apoptotic cells, particularly following chemotherapy or IO, may serve as a mechanism to suppress immune responses to dying tumor cells (171). MDSCs promote tumor progression by enhancing angiogenesis and metastasis via STAT3-driven VEGF upregulation, MMP-9 expression (57, 172, 173), and epithelial-mesenchymal transition (EMT) with TGF-β, IL-6, and IL1-β (172, 173) and prepare pre-metastatic niches in through mechanisms involving exosomes, TGF-β, S100A8/A9, and VEGF (58, 169, 172, 173). Reducing MDSC accumulation in TME can enhance immune responses, and this could be achieved by targeting cytokine pathways with inhibitors such as JAK, BTK, PI3K, and TKIs or using antibodies to neutralize activating cytokines (164).

M2-polarized TAMs can directly eliminate cytotoxic T-cells, induce the production of Tregs, and secrete immunosuppressive cytokines (174). Predominance of M2-polarized TAMs in the stroma of bladder tumors is associated with poor prognosis and resistance to Bacillus Calmette–Guérin (BCG) therapy (175). In the TME, TAM survival depends on the colony-stimulating factor (CSF)-1/CSF1R pathway; blocking CSF1/CSF1R has been proven to significantly reduce macrophage recruitment and M2 polarization and induce activation of CD8+ T-cells, thereby sensitizing tumor to ICI and prolonged survival in several other cancer types (176). TAMs also activate tumor cells through a mutagenic environment, pro-inflammatory mediators, and transcription factors like STAT3 and NF-κB (45). Additionally, TAMs influence PD-1/PD-L1 interactions and shifting them to immune-activating states may overcome resistance to PD-1/PD-L1 therapy, improving treatment outcomes (45, 177–180). PI3K inhibitors like CYH33 promote CD8+ and CD4+ T-cell infiltration while reducing M2 macrophages and Tregs (164).

In the TME, CD56^bright^CD16^-^ NK cells, the primary NK subtype in TME, can release VEGF, placenta growth factor, and IL-8/CXCL8, promoting angiogenesis and potentially supporting tumor growth (181). In the TME, factors like hypoxia, nutrient deprivation, and TGF-β hinder NK activity, weakening immune defense by fostering Treg formation (9, 182–184).

Bregs suppress immunity via IL-10, TGF-β, and PD-L1, encouraging Treg formation and dampening T-cell proliferation (66, 185–187). PD-L1 further enhances Breg-mediated suppression, reducing T-cell proliferation and affecting responses to immunogenic chemotherapy (188–190). While some tumor-infiltrating B-cells promote metastasis with co-expression of PD-L1 and IL-10, others—particularly granzyme-B-producing B-cells—may boost post-chemotherapy immune responses (64, 190).

CAFs, which produce growth factors and ECM, support cancer cells and induce chemoresistance and angiogenesis through VEGF-A, IL-6, TGF-β, and MMP-9 (191–194). Their metabolic adaptations, such as aerobic glycolysis driven by hypoxia and TGF-β, repurpose nutrients to sustain cancer growth and immune suppression (191, 195–198).

TGF-β activates CAFs, which enhance extracellular matrix protein expression, limiting T-cell infiltration into the TME. Recent studies link TGF-β to TME regulation by enhancing extracellular matrix deposition, promoting angiogenesis, and suppressing anti-tumor immune responses. Also, TGF-β inhibits DC and NK cell function, blocking T-cell activation, inducing immune exhaustion in CD8+ T-cells, and promoting Tregs. Additionally, TGF-β directly restricts CAR-T cells. Blocking TGF-β signaling in immune cells has shown potential for enhancing antitumor responses. However, targeting TGF-β is challenging due to its essential functions in normal tissues, necessitating further research on its immunosuppressive role in the TME (69, 110–112).

Adipocytes in the TME drive cancer progression by releasing lipids, cytokines, and adipokines that influence signaling, movement, and metabolism, supporting tumor growth. Their signaling further promotes angiogenesis and EMT marker expression, which are crucial for metastasis (199–201).

Beyond blood transport, endothelial cells in the TME interact with tumor cells, immune cells, fibroblasts, and ECM. This cross-talk regulates angiogenic factors (202) like VEGF that boost cancer cell aggressiveness and immunosuppression and influence TSC phenotype, EMT, ECM remodeling, and cancer cell intravasation (203).

TSCs play a central role in progression, metastasis, and chemoresistance. Their self-renewal ability is sustained by interactions with the TME, where factors like IL-6, TGF-β, and HIF-1 promote proliferation, angiogenesis, and survival, underscoring the complex interplay driving cancer dynamics (204–206).

### Metabolic barriers, TME-driven resistance and strategies to overcome

Metabolic barriers are another important part of TME-driven resistance. The high metabolic activity of cancer cells creates hypoxia, which induces HIF-1α, driving tumor angiogenesis and invasion (207). The high metabolic activity of cancer cells creates hypoxia, which induces HIF-1α, driving tumor angiogenesis and invasion. The adenosinergic pathway, regulated by hypoxia and TGF-β, suppresses immune responses, with adenosine inhibiting immune cell function and contributing to T-cell exhaustion (208, 209).

The Warburg effect shifts cancer metabolism toward anaerobic glycolysis, leading to lactate accumulation, acidosis, mitochondrial dysfunction, and glucose depletion. This upregulates exhaustion markers like PD-1, T cell immunoglobulin and mucin domain-containing protein 3 (TIM-3), and l ymphocyte-activation gene 3 (LAG-3) in T-cells, reducing their function and creating an immunsuppresive TME. However, at low concentrations, lactate can serve as an energy source for immune cells, presenting an opportunity to enhance cancer IO (210, 211). On the other hand, ICIs can restore T-cell function by increasing glucose in the TME, and blocking PD-L1 on tumors reduces their glycolysis, enhancing T-cell activity (212).

Beyond glucose, reduced availability of arginine and cystine also impairs T-cell function. Arginine depletion by MDSCs reduces T-cell proliferation, while cystine, crucial for T-cell antioxidative defense, is competitively consumed by tumor cells, further weakening immunity (213, 214).

Fatty acids are vital for immune cell function beyond energy production. In the TME, where acidification, hypoxia, and energy depletion occur, they support T-cell activity. Short-chain fatty acids enhance CAR-T cell efficacy, highlighting the therapeutic potential of targeting fatty acid metabolism in IO (215).

## Targeting the TME with immunotherapy in genitourinary cancers

### Kidney cancer

In 2021, an estimated 76,080 Americans were diagnosed with kidney and renal pelvis cancers, resulting in 13,780 deaths. RCC, which includes 85% of kidney tumors, with 70% being ccRCC (216), is the third most common urologic cancer. About 30% of RCC cases are diagnosed at a metastatic stage, severely impacting prognosis. Treatments, including antiangiogenic agents and targeted IO, have evolved, yet long-term responses remain rare due to high resistance. New treatment strategies and drugs are actively being evaluated in ongoing trials to improve outcomes.

Despite this, long-term responses are uncommon due to a high rate of resistance. ccRCC often involves VHL gene mutations, promoting angiogenesis via HIF-regulated VEGF (217).RCC’s immune landscape includes diverse T-cells, NK cells, B-cells, macrophages, and DCs. An immune atlas for RCC revealed varied T-cell and macrophage types, some linked to poor outcomes, indicating potential therapeutic targets (218). T-cell analysis showed that immune checkpoint expressions like PD-1, LAG-3, and Tim-3 correlate with more aggressive RCC forms (219). B-cells have a complex role; in ccRCC, their density is associated with a worse prognosis, potentially by influencing other immune cells through cytokine release (220).

Although RCC is immunogenic, it induces immune dysfunction by attracting suppressive cells like Tregs and MDSC to TME, hindering anti-tumor responses. PD-1/PD-L1 therapies have shown significant promise in altering the TME, becoming key in advanced RCC treatment (78). Nivolumab, particularly, has been effective not only in improving overall survival (OS) but also in modulating the TME by increasing the presence of CD4+ and CD8+ T-cells and chemokines like CXCL9 and CXCL10, which are linked to IFN-γ production. This demonstrates ICIs’ broader role in enhancing anti-tumor immunity by modulating the TME (221).

The dense microvascular network and recruitment of MDSCs, Tregs (222–225), and inhibiting DC maturation, driven by VEGF and VHL loss, promote an immunosuppressive TME but also serve as a target, such as VEGF inhibitors (226). Sunitinib inhibits this angiogenic activity and promotes lymphatic vessel formation, facilitating immune cell infiltration (227). These VEGF-targeting TKIs are key in metastatic RCC treatment but may lead to resistance. Inhibiting VEGF with ICI combinations can reduce this resistance (228, 229).

A spatial transcriptomics study on ccRCC demonstrated that TLS within the TME is crucial for developing anti-tumor antibody-producing plasma cells, and these plasma cells are disseminated into tumor tissue along fibroblastic tracks. These TLS-positive tumors were associated with improved PFS in ccRCC patients treated with ICIs (68). Another study on ccRCC indicates that memory B cells and plasma cells within TLS collaborate with other immune components to modulate T-cell functions. They act as antigen-presenting cells and secrete cytokines such as TNF and IFNγ, which help recruit additional immune cells. The presence of switched memory B cells in ICI responders suggests their potential role in producing anti-tumor antibodies, thereby potentially enhancing T-cell responses after ICI therapy for ccRCC (69).

A phase-I/II clinical trial evaluated IL-21 in combination with sorafenib, demonstrating antitumor activity and an acceptable safety profile in previously treated metastatic RCC patients. Most toxicities were grade I/II, with grade III skin rash as the only dose-limiting toxicity. These findings suggest that IL-21 is a promising candidate for further IO combinations in metastatic RCC (230). However, another study with recombinant IL-21 was terminated due to a low-tolerated dose of IL-21 (231).

For non-clear cell RCC (non-ccRCC) subtypes, anti-PD-1 monotherapy or its combinations with Ipilimumab or VEGF-targeted treatments therapies have shown promise due to the expression of PD-1/PDL-1 within the TME, despite these patients often being excluded from major trials (232, 233).

The preferred treatment for metastatic ccRCC involves a combination of an ICI and a VEGF-TKI. Treatment decisions are guided by the risk stratification provided by the International Metastatic Renal Cell Carcinoma Database Consortium (IMDC) criteria, also known as Heng criteria, which assess time from diagnosis to therapy initiation, performance status, hemoglobin, neutrophil and platelet counts, and serum calcium levels. Patients in the favorable-risk group, showing no prognostic factors, with a low disease burden, may be managed with active surveillance. Conversely, those in the poor-risk group—characterized by three to six prognostic factors—should receive systemic therapy with either an ICI and VEGF-TKI combination or a dual IO regimen (234, 235).

Beyond PD-1/PD-L1, other checkpoints like TIGIT, LAG-3, TIM-3, and ILT-4 are being investigated as ICI. In RCC, phase-Ib/II trials are testing anti-LAG3 Favezelimab, anti-ILT-4 MK-4830, and anti-TIGIT Vibostolimab with Pembrolizumab for advanced ccRCC (236, 237).

In a phase-I/II trial with advanced malignancies including RCC, Ieramilimab, anti-LAG3 antibody, combined with Spartalizumab, anti-PD-1 antibody. However, no responses were observed with Ieramilimab alone, and only modest antitumor activity was seen with the combination treatment. Treatment-related adverse events (TRAEs) occurred in 56% of single-agent and 69% of combination therapy patients, mostly mild fatigue, gastrointestinal issues, and skin disorders. Serious TREA occurred in 5% of patients in the single-agent group and 5.8% in the combination group (238, 239). A phase-I three-arm trial is also assessing Tobemstomig (anti-PD-1/LAG3 bispecific antibody) and Tiragolumab (anti-TIGIT) with Pembrolizumab plus Axitinib as the control (240). Recruitment for CDX-585 (PD-1/ILT-4 bispecific antibody) in advanced solid tumors, including urogenital neoplasms, is ongoing (241).

While IO has mainly focused on checkpoint inhibition, other IOs, like oncolytic viruses and CAR-T cells, are emerging in RCC. CAR-T cells engineered for antigen recognition show promise, especially when combined with TKIs or radiotherapy (217, 242, 243). CD70-CD27 signaling may promote tumor growth by limiting T-cell expansion and enhancing Tregs with high CD70 expression in tumors like ccRCC. The first complete response in metastatic ccRCC was seen with CTX130, a CD70-targeted CAR-T cell, achieving an 81.3% disease control rate. However, grade 1/2 cytokine release syndrome (CRS) occurred in 50% of patients with no grade ≥3 cases, and serious adverse events (AEs), which were all due to CRS, were reported in 25% of the patients (244). ALLO-316, another CD70-targeted CAR-T, reached a 100% control rate in the phase-I TRAVERSE trial. Common AEs were fatigue (71%), nausea (61%), CRS (58%, mostly low grade, with one grade 3 case [3%]), neutropenia (55%), leukopenia (45%), and anemia (45%) (245). Ongoing phase-I trials with CGC729, an anti-CD70 CAR-NKT cell, showed preliminary evidence for tumor response in both CD70 positive and negative tumors. No dose-limiting toxicity occurred, and common AEs were neutropenia, thrombocytopenia, and leukopenia—with no cytopenia above grade 2 (246, 247). On the other hand, anti-VEGFR CAR-T cells demonstrated no objective responses in metastatic settings in a terminated phase-I/II trial (248). Still, CAR-T cells targeting alternative markers such as carbonic anhydrase IX (CAIX), a TAA and cell surface protein that is overexpressed in many types of cancers including ccRCC, is ongoing (249, 250). CAR-T-cells are also under investigation, with promising preclinical evidence suggesting benefits when combined with TKIs or RT in RCC (217, 242, 243).

BsAbs targeting RCC focus on immune checkpoints and tumor markers. AK104 (cadonilimab), a PD-1×CTLA-4 BsAb, plus lenvatinib showed encouraging anti-tumor activity and a manageable safety profile for previously immunotherapy-treated ccRCC (251), and a trial with a combination of cadonilimab and axitinib as a first-line treatment for mRCC is ongoing (252).

Recent research showed that ENPP3 mRNA is highly expressed in ccRCC. *In vitro* models suggest ENPP3 as a promising anti-ENPP3 BsAb target for ccRCC (104). An ongoing study is exploring ENPP3 and CD3 BiTE (XmAb819) treatment in advanced ccRCC (253). Another study is exploring targeting HER2/HER3 via MCLA-128 BsAb in advanced NRG1-fusion-positive RCC (254).

On the vaccine front, different strategies aim to heighten tumor neoantigens, focusing on DNA/RNA-based, peptide-based, and cell-based vaccines to the immune system, notably enhancing the priming phase of T-cells (217, 243). A phase-I trial showed that the GEN-009 neoantigen vaccine combined with anti-PD-1 therapy can induce strong, specific immune responses with low toxicity, with AEs limited to injection site reactions, mild myalgia, and fatigue (255). Additionally, the NeoVAX personalized cancer vaccine, combined with Ipilimumab, is currently under investigation for stage III-IV ccRCC (256), though a separate trial of a personalized cancer vaccine with standard treatment was terminated due to low enrollment (257).

Another phase-II trial is testing an autologous DC/peptide vaccine targeting antigens associated with tumor blood vessels, combined with cabozantinib to inhibit angiogenesis, induce the maturation and organization of tumor vasculature and promote the development of TLS that facilitates specific T-cell activation in localized ccRCC patients (258).

While the primary challenge of immuno-oncology therapies is immune-related AE, most of these novel treatments have demonstrated a tolerable safety profile. However, the high production costs associated with cancer vaccines and CAR-T cell therapies suggest that integrating these modalities into clinical practice may take time.

Selected completed and ongoing IO studies for RCC and their targets in the TME are summarized in **Table 1**.

**Table 1:** Selected completed and ongoing IO studies for RCC and their targets in the TME.

Study/NCT Number	Phase	N	Therapy	Target in TME	RCC Type	M status	Primary Endpoint	Therapy Setting	Status
**NCT05127824** (394)	II	42*	Autologous DC Vaccine in Combination +Cabozantinib (RTK/VEGF inhibitor)	increasing DC and eff T-cells + inhibiting angiogenesis	ccRCC	M0	Safety and immune response	Neoadjuvant	Active, recruiting (Start: July 2023)
**NCT02950766** (395)	I	19	Personalized NeoAntigen Cancer Vaccine +Ipilimumab (Anti-CTLA-4)	Increasing APC +increased T-cell proliferation in LN	ccRCC	M0 or M1 with NED	DLT	Adjuvant	Active, not recruiting (Start: March 2019)
**CheckMate214** **(NCT02231749)** (396)	III	1390	Nivolumab (Anti-PD-1) +Ipilimumab (Anti-CTLA-4)	Increasing eff T-cells +increased T-cell proliferation in LN	ccRCC	Locally advanced M0 or M1	ORR, OS, PFS	Metastatic	Active, not recruiting (Start: October 2014)
**JAVELIN Renal 101** **(NCT02684006)** (397)	III	888	Avelumab (Anti-PD-L1) +Axitinib (RTK/VEGF inhibitor)	Increasing eff T-cells + inhibiting angiogenesis	ccRCC	Locally advanced M0 or M1	PFS, OS	Metastatic	Active, not recruiting (Start: March 2016)
**NCT04626479** (236)	I/II	400*	Favezelimab (Anti-LAG3) +Pembrolizumab (Anti-PD-1)	Increasing eff T-cells	ccRCC	Locally advanced M0 or M1	AE and ORR	Metastatic	Active, not recruiting (Start: December 2020)
**NCT05805501** (240)	II	210*	Tobemstomig (anti-PD-1/LAG3 bispecific antibody) +/- Tiragolumab (Anti-TIGIT) +/- Axitinib (RTK/VEGF inhibitor)	Increasing eff T-cells +/- inhibiting angiogenesis	ccRCC	Locally advanced M0 or M1	PFS	Metastatic	Active, not recruiting (Start: April 2023)
**NCT02830724** (398)	I/II	124*	Anti-CD70 CAR-T cells	Increasing tumor specific T-cells	CD70+ ccRCC	M0 unresectable or M1	AE and response rate	Unresectable/metastatic	Active, recruiting (Start: April 2017)
**NCT06182735** (399)	I	9*	Anti-CD70 CAR-NKT Cells (CGC729)	Increasing tumor specific NKT-cells	ccRCC	M1	DLT	Metastatic	Active, recruiting (Start: July 2023)
**NCT03633110** (400)	I/II	24	GEN-009 neoantigen vaccine +PD-1 inhibitors	Increasing APC and tumor specific eff T cells	RCC	M1	AE and T-cell response	Metastatic	Completed (February 2022)
**NCT04969354** (250)	I	20*	Anti-CAIX CAR-T cells	Increasing tumor specific T-cells	RCC	advanced M0 or M1	AE, ORR	Unresectable/Metastatic	Active, recruiting (Start: October 2021

N, Patient number; M, Metastasis; eff T cell, effector T-cell; DC, Dendritic cell; NKT cell, Natural killer T-cell; APC, Antigen presenting cell; ccRCC, clear cell Renal Cell Carcinoma; non-ccRCC, Non-clear cell Renal Cell Carcinoma; LN, Lymph node; ORR, Objective Response Rate; PFS, Progression-Free Survival; OS, Overall Survival; RTK, Receptor tyrosine kinase; VEGF, Vascular endothelial growth factor; DLT, Dose limiting toxicity; AE, Adverse event; *Estimated number.

### Prostate cancer

Prostate cancer ranks as the second-most common and sixth deadliest cancer among men. Treatments like surgery and radiotherapy are effective for localized cases, but around 20% to 30% of patients will experience recurrences. Androgen deprivation therapy (ADT) is the main treatment for metastatic prostate cancer, though it often leads to resistance and the development of castration-resistant prostate cancer (CRPC), which can be non-metastatic or metastatic. CRPC adapts through androgen synthesis, receptor modifications, and cellular changes like EMT. Despite new therapies like second-generation antiandrogens and taxanes, CRPC typically remains incurable, with treatments extending survival only slightly (259).

Prostate cancers, with low mutational burden and an immunosuppressive TME, show resistance to IO. Though solid tumors with dMMR respond well to IO (260), the prevalence of dMMR is lower than 3% in prostate cancer (261). Dense CD8+ T-cell infiltration is associated with better outcomes in high-risk localized prostate cancer. However, TME interactions, driven by IL-6 and IL-8, promote suppressive cells like PMN-MDSCs and M2-TAMs, contributing to worse outcomes (262–266). CAFs are abundant in prostate cancer, contributing to immune evasion and therapy resistance by inhibiting CD8^+^T-cell function and inducing CTLA-4 overexpression (259). Stroma-epithelial interactions influenced by fibroblast growth factors, and TGF-β signaling further drive tumor progression and immune suppression (260). Additionally, toll-like receptors (TLRs), particularly TLR4 and TLR9, have been implicated in prostate cancer invasion and metastasis (267).

A study on radical prostatectomy specimens revealed that the presence of TLS is positively associated with MHC signatures, as well as T-cell and B-cell cluster signatures, and negatively associated with immune suppressive signature (267). Another study on prostate cancer demonstrated that TLS is present at various stages of cancer progression, and dynamic changes influence their functionality in the TME. Specifically, COX2 and Treg were found to inhibit TLS-driven tumor immunity. Given this, COX2 and Treg emerge as promising therapeutic targets to enhance TLS-driven tumor immunity. Additionally, the presence of HEV and lymphatics in prostate tissue suggests that TLS could effectively serve as a platform for delivering cell-based and/or COX2-inhibiting therapies, potentially leading to more effective management of prostate cancer (268).

In 2010, sipuleucel-T became the first IO approved for metastatic castration-resistant prostate cancer (mCRPC) with minimal metastasis. It uses autologous mononuclear cells activated ex vivo to stimulate cytotoxic T-cells and targets prostatic acid phosphatase to stimulate an immune response. A phase-III trial showed that it extended survival by over 4-months and reduced mortality by 22% in advanced prostate cancer, though it resulted in minimal tumor shrinkage. AEs were similar in patients receiving sipuleucel-T (98.6%) and placebo (96.1%).Despite not impacting progression-free survival (PFS), the modest success of sipuleucel-T indicates the potential for future use of ICI in prostate cancer treatment. However, trials combining Sipuleucel-T with other therapies were halted due to logistical challenges (269, 270).

ICI in mCRPC has shown mixed results. Ipilimumab provided a slight survival benefit post-RT/chemotherapy, suggesting potential TME modulation (271). However, its combination with Nivolumab resulted in significant side effects, with grade 3-4 AEs occurring in approximately 42%-53% of patients and four treatment-related fatalities (272). On the other hand, PD-1/PD-L1 inhibitors like Pembrolizumab showed better efficacy with hormonal therapy, impacting the TME in select cases (273), while combinations like Atezolizumab with hormonal therapy failed to improve survival (274), emphasizing the need for targeted approaches (269, 275–277).

Novel checkpoint targets, such as B7-H3, LAG-3, 4-1BB, and TIGIT, are being heavily investigated in prostate cancer (265). Enoblituzumab, an anti-B7-H3 antibody, has been tested in high-risk localized prostate cancer and demonstrated promising clinical activity, with 12% of patients experiencing grade 3 AEs and no grade 4 AEs reported (278, 279). Additionally, a novel anti-LAG-3/TIGIT bispecific IgG4 antibody, ZGGS15, demonstrated anti-tumor efficacy in *in-vitro* models, with human trials forthcoming (280).

Radiotherapy may enhance ICI efficacy in prostate cancer by boosting antigen presentation and T-cell infiltration (281). A phase-I study combined nivolumab with brachytherapy and external beam radiation for grade group-5 prostate cancer patients. This combination was well tolerated and associated with evidence of increased immune infiltration and antitumor activity (282). A phase-III study assessed the therapeutic impact of ipilimumab combined with RT in mCRPC patients. A preplanned long-term analysis demonstrated a significant improvement in overall survival with this combination. The 5-year OS rate was approximately two to three times higher compared to those receiving RT alone (271).

CAR-T-cells targeting the prostate-specific membrane antigen (PSMA) with CD28 co-stimulation have shown enhanced *in-vivo* anti-tumor effects, promising for CRPC treatment. A phase-I clinical trial (283) tested a PSMA-targeted CAR-T-cell therapy in CRPC to counter the immunosuppressive environment enriched with TGF-β (262). It has resulted in a high response rate but treatment failure with upregulation of TME-sourced inhibitory signals. AEs included grade ≥2 CRS in five of 13 patients, with one fatality due to grade 4 CRS and concurrent sepsis (284).

BiTE therapies targeting PSMA and CD3, such as Pasotuxizumab, show promise in metastatic mCRPC. Pasotuxizumab led to dose-dependent tumor responses in a phase-I trial, with three patients achieving ≥50% PSA reduction and one showing complete regression of soft tissue metastases, albeit all patients had AEs, mostly fever (94%), chills (69%), and fatigue (50%). Grade ≥3 AEs occurred in 81%, mainly decreased lymphocytes and infections (44%), and no grade 5 adverse event was seen (285, 286). Another PSMA-CD3 BiTE, JNJ-081, led to transient PSA reductions without radiographic response, showing a tolerable safety profile. Dose-limiting toxicities occurred in 10.3% of patients, and CRS was observed only at higher doses. No treatment-related deaths were reported (287). Though early BiTE studies show limited efficacy, they highlight PSMA’s potential as a target for BiTE despite the high adverse event rates in some trials.

Tarlatamab, a BiTE targeting DLL3, showed clinical activity in neuroendocrine prostate cancer with a 10.3% objective response rate (ORR) and median PFS of 1.9 months. All patients had AEs, with no fatalities. Common AEs were CRS, mostly grade 1–2 (65.0%), pyrexia (52.5%), and dysgeusia (42.5%), and treatment discontinuation was low (7.5%) (288). Additionally, JNJ-902, a BiTE targeting CD3 and TMEFF-2, led to PSA declines in 8 of 72 patients with minimal dose-limiting toxicity. Fatigue (45%) and decreased appetite were the most common AEs. Dose-limiting toxicities occurred in 2.7% of patients, while CRS occurred in 5.5%, resolving within 2–3 days (289). Other BiTE in trials include Xaluritamig (anti-STEAP1), LAVA-1207 (anti-Vδ2), JNJ-78278343 (anti-KLK2), and REGN5678 (anti-CD28 and anti-PSMA), each in phase-I trials (290–293). Though BiTEs face inherent limitations in efficacy in mCRPC due to the cold TME, characterized by low T-cell infiltration, and further complicated by ADT that deregulate intratumoral T-cells (105).

A phase-II trial evaluated the combination of pembrolizumab and anti-CD3 x anti-HER2 Bispecific Antibody-Armed Activated T-Cells (HER2-BAT) in mCRPC showed promising results, with 5 of 14 patients achieving 6-month PFS, a median PFS of 5 months, and overall survival of 31.6 months. PSA levels dropped in six patients, and 38.5% remained progression-free (294). Another study is exploring targeting HER2/HER3 via MCLA-128 BsAb in advanced NRG1-fusion-positive prostate cancer (254).

Prostate stem cell antigen (PSCA) is also a promising target as IO in prostate cancer, particularly in metastatic and treatment-resistant forms, as it is not present in normal prostate cells (295). PSCA-specific CAR-T-cells (BPX-601) showed biological activity in mCRPC with a PSA decline of over 30%, and radiographic improvements were seen in 4 of 14 patients. Some patients also showed activation of peripheral blood CAR and endogenous T-cells, increased TCR diversity, and changes in the TME. The most common grade ≥3 adverse event was myelosuppression. All patients had CRS. Immune-effector cell-associated neurotoxicity syndrome occurred in 25.0% of patients and resolved. 12.5% had dose-limiting toxicity of fatal neutropenic sepsis (296, 297). Additionally, a new trial demonstrated the anti-cancer activity of PSCA-targeted CAR-T cells in mCRPC with no dose-limiting toxicity. CRS (Grade 1 or 2) occurred in only 35.7% of treated patients (298). These studies highlight the potential of targeting cancer antigens in TME.

Some trials are testing poxvirus-based cancer vaccines, PROSTVAC-V and PROSTVAC-F, targeting prostate-specific-antigen (PSA) with immune stimulants. A phase-II trial showed an 8.5-month median survival increase, with injection site reactions as the most common AEs and some systemic effects like fatigue, fever, and nausea. However, a larger phase-III trial (NCT01322490) did not confirm this finding (269). An ongoing trial is assessing PROSTVAC combined with a monoclonal antibody for recurrent prostate cancer (299). Furthermore, intradermal administration of the telomerase-based cancer vaccine GX301 has shown immune activation within prostate tissue and survival benefits in mCRPC patients. Panniculitis-like inflammation at the injection site was the most common side effect and increased with vaccine doses. Systemic side effects were rare and mostly unrelated to GX301 (300, 301).

Recognizing the role of TLRs in prostate cancer, various trials are exploring TLR-targeted therapies. For example, intratumoral administration of SD-101, a TLR-9 agonist, with or without Pembrolizumab in oligometastatic prostate cancer patients undergoing radiotherapy is under phase-II investigation (302). Also, Mobilan (M-VM3), a recombinant adenovirus-based gene therapy, has shown tumor responses in early-stage prostate cancer through stimulation of TLR5 signaling and consequent immune activation following intra-prostatic injection, accompanied by temporary PSA and cytokine (G-CSF, IL-6) increases and greater lymphoid infiltration in prostate tissue, unlike in placebo patients. Mobilan was safe and well-tolerated at all doses, with no identified maximum tolerated dose. The most common adverse event was abnormal laboratory values (303).

Despite advancements in IO for prostate cancer, more research is needed to fully understand interactions in the TME. While ICIs show promise, the effectiveness of combining ICIs or pairing them with conventional treatments remains underexplored (262).

**Table 2** summarizes selected IO studies for prostate cancer and their targets within the TME.

**Table 2:** Selected completed and ongoing IO studies for prostate cancer and their targets in the TME.

Study/NCT Number	Phase	N	Therapy	Target in TME	Prostate Cancer Type	M status	Primary Endpoint	Therapy Setting	Status
**NCT02923180 (** 279)	II	33	Enoblituzumab (MGA271) (Anti-B7-H3)	Increasing eff T-cells	Intermediate and high-risk localized prostate cancer	M0	AE and efficacy	Neoadjuvant	Active, not recruiting (Start: February 2017)
(303)	I	24	Mobilan (Adenovirus-based gene therapy)	Stimulation of TLR5 and consequent immune activation	Early-stage prostate cancer	M0	Safety and tolerability	Adjuvant	Completed
**NCT03315871** (401)	II	29	Prostvac + CV301+ MSB0011359C	Increasing T-cells against PSMA positive cells	Recurrent or metastatic prostate cancer	M0/M1	%30 decline in PSA	Biochemically recurrent non-metastatic	Active, not recruiting (Start: March 2018)
**COSMIC-021** **(NCT03170960)** (402)	I/II	1732	Cabozantinib (RTK/VEGF inhibitor) +Atezolizumab (Anti-PD-L1)	Inhibiting angiogenesis, converting M2 to M1 and reducing MDSC and Tregs + increasing eff T-cells	CRPC	Locally advanced M0/M1	MTD, ORR	Metastatic	Active, not recruiting (Start: September 2017)
**CheckMate 650** **(NCT02985957)** (403)	II	351	Nivolumab (Anti-PD-1) + Ipilimumab + (Anti-CTLA-4)	Increasing eff T-cells +increased T-cell proliferation in LN	CRPC	M1	ORR, rPFS	Metastatic	Active, not recruiting (Start: March 2017)
**IMbassador250** **(NCT03016312)** (404)	III	772	Atezolizumab (PD-L1 inhibitor) + Enzalutamide (ARI)	Increasing eff T-cells + decreasing prostate cancer cells	CRPC	M1	OS	Metastatic	Completed (December 2022)
**BAY2010112** **(NCT01723475)** (405)	I	47	Pasotuxizumab (PSMA-targeting BiTE)	Increasing T-cells against PSMA+ cells	CRPC	M1	Adverse events and MTD	Metastatic	Completed (September 2018)
(287)	I	39	JNJ-081 (PSMA-CD3 BiTE)	Antitumor activity to PSMA-expressing tumor cells by activating CD3-expressing T-cells	CRPC	M1	Safety and anti-tumor response	Metastatic	Completed
**NCT04221542 (** 290)	I	261*	Xaluritamig- AMG 509 (STEAP1-CD3 BiTE)	Antitumor activity to STEAP1-expressing tumor cells by activating CD3-expressing T-cells	CRPC	M1	AE and DLT	Metastatic	Active, recruiting (Start: March 2020)
**NCT05369000** (291)	I/II	180*	LAVA-1207 (PSMA-Vδ2 BiTE) +/- IL2 +/-Pembrolizumab (Anti-PD-1)	Antitumor activity to PSMA-expressing tumor cells by activating Vδ2 expressing T-cells	CRPC	M1	AE and DLT	Metastatic	Active, recruiting (Start: June 2022)
**NCT04898634** (292)	I	260*	JNJ-78278343 (KLK2-CD3 BiTE)	Antitumor activity to KLK2-expressing tumor cells by activating CD3-expressing T-cells	CRPC	M1	DLT	Metastatic	Active, recruiting (Start: July 2021)
**NCT03972657** (293)	I/II	345*	REGN5678 (PSMA-CD28 BiTE) +/- Cemiplimab (Anti-PD-1)	Antitumor activity to PSMA-expressing tumor cells by activating CD28-expressing T-cells	CRPC	M1	AE, DLT and ORR	Metastatic	Active, recruiting (Start: August 2019)
**EudraCT 2014-000095-26** (301)	II	120	Telomerase-based cancer vaccine (GX301)	Inducing telomerase-specific T-cells	CRPC with response/disease stability after docetaxel	M1	Safety and immunological response evaluation	Metastatic	Completed
**NCT03873805** (297)	I	14	PSCA-CAR-T cells	Increasing tumor specific CAR-T cells	PSCA-positive CRPC	M1	Safety and DLT	Metastatic	Active, not recruiting (Start: August 2019)

N, Patient number; M, Metastasis; eff T cell: effector T cell; CRPC, Castration-Resistant Prostate Cancer; RT, Radiotherapy; ORR, Objective Response Rate; rPFS, Radiographic Progression-Free Survival; OS, Overall Survival; OSR, Overall Survival Rate; MTD, maximum tolerated dose; PSMA, Prostate-specific membrane antigen; PSA, Prostate-specific antigen; PSCA, prostate stem cell antigen; ARI, Androgen receptor inhibitor; RTK, Receptor tyrosine kinase inhibitor; BiTE, Bispecific T-cell engagers; DLT, Dose-limiting Toxicity; TLR, Tall-like receptor; AE, Adverse events; *Estimated number.

### Bladder cancer

Bladder cancer is the 10th most common cancer globally, with over 500,000 new cases annually. About 75% of cases are non-muscle-invasive (NMIBC), confined to the mucosa, while the rest are MIBC or metastatic (304, 305).

The bladder cancer TME includes stromal cells, CAFs, immune cells, and ECM components, which actively influence cancer progression and drug resistance (306). Cytotoxic CD8+ T-cells are linked to improved outcomes in bladder cancer and are crucial for the effectiveness of IO therapies like anti-PD-1/PD-L1, though they can become exhausted. DC presence, particularly cDC, improves treatment response, such as with BCG therapy. Additionally, TAMs, especially M2-type and MDSCs, promote immune suppression and tumor progression, correlating with poorer outcomes (307).

The stroma affects treatments like intravesical-BCG, ICIs, chemotherapy, and trimodality therapy, which combines transurethral resection of the tumor, chemotherapy, and radiotherapy (306). Intravesical-BCG binds to fibronectin on tumor cells, causing apoptosis, necrosis, and cytokine release, enhancing immune response. BCG activates macrophages and T-cells, boosting cytokine-mediated reactions. Studies suggest that BCG upregulates PD-L1 in bladder cancer via the MAPK pathway, contributing to immune evasion and potential BCG failure. Combining BCG with anti-PD-L1 therapy enhances CD8+ T-cell infiltration, reduces MDSCs, and improves tumor suppression. Higher PD-L1 expression in BCG responders suggests its role in treatment resistance, highlighting the potential of combination therapy, though further validation is needed (308).

Intravesical BCG is the main treatment for high-risk NMIBC, reducing recurrence and improving survival, with maintenance therapy for intermediate to high-risk cases. BCG-refractory patients often face radical cystectomy, which significantly impacts their quality of life. Therefore, there’s interest in augmenting BCG therapy with other treatments, such as IFN-α, recombinant adenovirus IFN-α, or a mix of IFN-α, IL-2, and GM-CSF (309).

Pembrolizumab is FDA-approved for BCG-refractory NMIBC in patients unable or unwilling to undergo cystectomy (310). While PD-L1 expression was initially a predictive marker for therapy response, its reliability is limited. Studies now focus on CD8+ T-cell signatures and tumor mutation burden as better predictors (307). Ongoing trials, including Durvalumab with BCG, aim to optimize NMIBC treatment by targeting TME components (310).

Genomic analysis identifies BC subtypes, with “basal/squamous” showing high IO responsiveness due to its immune marker profile, while “luminal papillary” has less immune infiltration. Tumor pathways like Wnt-β catenin and PPAR-γ influence immune cell presence and IO resistance. FGFR3 mutations are linked to lower T-cell levels, but trials like IMVIGOR-210 and Checkmate-275 found no direct correlation with IO efficacy, possibly due to TGF-β signaling interactions (307, 311–313).

MIBC has a high mutation rate, impacting ICI response. Indications for atezolizumab and pembrolizumab now include MIBC patients ineligible for cisplatin, with high PD-L1 expression, or ineligible for any platinum chemotherapy, regardless of PD-L1 levels (314, 315). Over 20% of atezolizumab patients report common AEs like fatigue, decreased appetite, and nausea, with severe events in at least 2% including fatigue and urinary infections. Similarly, over 20% of pembrolizumab patients experience fatigue, musculoskeletal pain, and decreased appetite, with severe AEs like urinary tract infections, anemia, and fatigue occurring in at least 2% (316). Avelumab, approved for maintenance therapy post-chemotherapy in metastatic urothelial carcinoma (mUC), shows 98.3% of patients experiencing AEs, 53.8% severe. TRAEs were noted in 78.2%, with 19.5% severe. After 12 months, 50% reported TRAEs, 11.9% severe, leading to a 10.2% discontinuation rate and one death from immune-mediated nephritis. Additionally, 22.9% developed immune-related AEs, with 4.2% severe (317).

B7-H3 (CD276) serves as an alternative immune checkpoint target, alone or in combination with PD-1 therapies. Enoblituzumab, an investigational anti-B7-H3 antibody, has an Fc domain that enhances antibody-dependent cellular cytotoxicity via Fcγ receptor interactions. In an early phase-I/II trial for advanced cancers, including urothelial cancer, combining Enoblituzumab with Pembrolizumab showed a limited response, with an ORR of 5.9%. TRAEs were reported in 87.2% patients, with 28.6% experiencing severe events (grade ≥3) (318, 319).

Growing evidence supports the immunomodulatory properties of RT and its potential to enhance outcomes when used in conjunction with ICI. A phase-II trial showed that durvalumab with RT followed by adjuvant durvalumab was safe with promising efficacy in patients with pure or mixed urothelial BC with unresectable tumors and were unfit for surgery or cisplatin. No dose-limiting toxicities were observed. The treatment was generally well-tolerated, with fatigue and diarrhea (likely radiation-related) being the most common TRAEs and there were no treatment-related deaths (320). Another phase-II study in patients with MIBC found that combining durvalumab and tremelimumab with concurrent RT is a feasible, safe, and effective approach, demonstrating promising response rates and bladder preservation. Grade 3 to 4 toxicities occurred in only 31% of patients, most commonly diarrhea and acute kidney failure, each affecting 6% (321). A phase-II trial in patients with locally advanced urothelial bladder cancer evaluated neoadjuvant radio-immunotherapy with nivolumab plus RT followed by radical cystectomy. The approach was found to be feasible and safe. While survival data remain immature, the 12-month disease-free survival (DFS) rate was 90.6%. TRAEs occurred in 54.5% of patients, mostly as grade 1-2. Common TRAEs included thyroid and gastrointestinal disorders (15.2% each) and skin reactions (33.3%). TRAEs led to discontinuation of treatment in 25.8% of patients (322).

CAR-T cell technology has shown promise in targeting BC, leveraging TAA such as HER2, MUC1, and EGFR, which are highly expressed in BC tissues. Preclinical studies, including PD-1/CAR-T cells targeting PD-1 ligands and MUC1 CAR-T cells demonstrating specific cytotoxicity against MUC1-positive BC cells, highlight its potential (323). Clinical trials of CAR-T with an oncolytic adenovirus, CAdVEC (324), or CAR-macrophage (325) cells targeting HER2 in advanced solid tumors, including BC, are currently underway.

BsAb treatments are emerging IO strategies for mUC. A phase-II trial in China studies a PD-1/LAG-3 BsAb (RO7247669) with or without tiragolumab, compared to Atezolizumab alone for platinum-ineligible mUC patients, showing a 17.1% ORR and manageable AEs. AEs were mild to moderate (grade 1-2). Severe TRAEs (grade 3) occurred in 17.1% of patients, with no cases of extremely severe AEs (grade 4-5) or dose-limiting toxicities (326, 327). A separate phase-II trial evaluated Disitamab vedotin (anti-HER2 antibody-drug conjugate) with cadonilimab (PD-1/CTLA-4 BsAb) in HER2-positive mUC, reporting a 75% ORR, 100% disease control rate, and tolerable side effects. Among the patients, 69.2% experienced TRAEs, the most common being increases in AST/ALT (30.8%), fever, and anemia (23.1%). Severe TRAEs (Grade 3 or higher) were reported in 15.4% of patients, including one death due to a cerebrovascular event. Furthermore, 30.8% experienced immune-related AEs, with 15.4% severe, including conditions like hepatitis and colitis. These findings suggest promising efficacy and safety, particularly for cisplatin-intolerant patients (328, 329). Also, a phase-I trial explored intravesical treatment of anti-EpCAM/CD3 BiTE (Catumaxomab) for high-risk NMIBC and had promising results with reduction in EpCAM+ urine cells and was well tolerated. Patients did not encounter any serious AEs related to the medication; however, urinary tract infections associated with the procedure were frequent (330).

ALT-803 (N-803), an IL-15 superagonist, shows promise for BCG-unresponsive high-grade NMIBC by activating NK cells, enhancing CD8+ T-cell activity, and avoiding Tregs stimulation, thereby boosting BCG-induced immune responses in the TME. Unlike ICIs, it reduces tumor burden and improves immune infiltration in preclinical models (331, 332). In the QUILT-3.032 trial, intravesical N-803 with BCG achieved a 71% complete response rate in carcinoma *in situ* CIS, 89.2% cystectomy avoidance, and 100% BC-specific survival at 24 months, with good tolerability. Most TRAEs in patients treated with BCG plus N-803 were mild to moderate (grades 1 to 2, 86%), and grade 3 immune-related AEs were seen in 3.66%. Therefore, ALT-803 offers a durable non-ICI option for PD-1/PD-L1-ineligible patients (333–335).

Intravesical viral particles that infect cancer cells to trigger immune responses or deliver therapies, such as weakened measles virus, IFN-α-inducing viruses, and attenuated Salmonella enterica. Another therapy, Nadofaragene firadenovec (Adstiladrin), is a gene therapy using non-replicating viral DNA vectors to produce INF-α2b in tumor cells, leading to apoptosis. It also induces MHC-I expression, enhancing T-cell activity against tumors (314, 336). In a phase-III trial, 53.4% of BCG-unresponsive CIS patients achieved complete response within three months, with 45.5% maintaining it at 12 months, showing a favorable safety profile. Most AEs among the patients were transient and mild to moderate (grade 1 or 2). Most adverse events among the patients were transient and mild to moderate (grade 1 or 2). Severe AEs (grade 3 or 4) occurred in 18% of the patients, with micturition urgency being the most common study drug-related grade 3–4 AE, affecting 1% (337, 338). FDA approved it for high-risk BCG-unresponsive NMIBC with CIS in 2022 (339).

CG0070, an oncolytic adenovirus expressing GM-CSF, targets mutated or deficient retinoblastoma tumor suppressor genes in BCG-unresponsive bladder cancer. It induces tumor cell lysis and boosts immune response via GM-CSF, offering a promising non-ICI approach. In a phase-III trial for patients with BCG-unresponsive carcinoma *in situ*, 53.4% achieved a complete response within 3 months of the initial dose, and 45.5% maintained this response at 12 months. Common TRAEs included urinary bladder spasms (36%), hematuria (28%), dysuria (25%), and urgency (22%). Immunologic AEs featured flu-like symptoms (12%) and fatigue (6%). Severe AEs (Grade 3) were limited to dysuria (3%) and hypotension (1.5%), with no Grade 4/5 AEs reported (340, 341). Additionally, combining this treatment with pembrolizumab showed a promising complete response rate of 87.5%, with transient, mild to moderate genitourinary TRAEs and no severe or serious AEs (332, 342).

Selected IO studies for bladder cancer and their targets within the TME are summarized in **Table 3**.

**Table 3:** Selected completed and ongoing IO studies for bladder cancer and their targets in the TME.

Study/NTC Number	Phase	N	Therapy	Target in TME	Bladder Cancer Type	M status	Primary Endpoint	Therapy Setting	Status
**NCT04452591** (341)	III	190*	Cretostimogene (CG0070) (oncolytic adenovirus expressing GM-CSF)	Targets mutated or deficient retinoblastoma tumor suppressor genes + induces tumor cell lysis and boosts immune response via GM-CSF	BCG-unresponsive BC	M0	CRR, EFS	Adjuvant	Recruiting (Start: October 2020)
**NCT01687244** (338)	III	157	Nadofaragene firadenovec	Using non-replicating viral DNA vectors to produce INF-α2b in tumor cells, leading to apoptosis, induces MHC-I expression and Increasing eff T-cells	BCG Unresponsive High-grade NMIBC	M0	CRR	Adjuvant	Completed (January 2016)
**NCT03022825** (334)	II/III	190	ALT-803 (N-803) (IL-15 super agonist) +BCG	Activating NK cells, enhancing eff T-cell activity + secretion of cytokines and local immune activation	BCG Unresponsive High-Grade NMIBC	M0	CR, DFR	Adjuvant	Active, not recruiting (Start: June 2017)
**KEYNOTE-057** **(NCT02625961)** (406)	II	320 (estimated)	Pembrolizumab (Anti-PD-1) +/- Vibostolimab (Anti-TIGIT) or +/- Favezelimab (Anti-LAG-3)	Increasing eff T cell	High-risk NMIBC (BCG unresponsive TCC and ineligible/refused RC)	M0	CR, DFS, AE	Adjuvant	Recruiting (Start: February 2016)
**NCT03740256** (324)	I	45*	CAdVEC (oncolytic adenovirus) + HER2-specific CAR-T-cells	CAdVEC will create a pro-inflammatory TME, promote the recruitment and expansion of transferred HER2-CAR-T-cells + CAR-T will attack HER2-positive tumor cells	HER2-positive BC	Locally advance M0/M1	DLT	Unresectable/Metastatic	Recruiting (Start: December 2020)
**IMVIGOR 210** **(NCT02108652)** (407)	II	310	Atezolizumab (Anti-PD-L1)	Increasing eff T cell	UC	Locally advance M0/M1	CR and PR	Unresectable/Metastatic	Completed (February 2023)
**NCT02475213** (319)	I	17	Enoblituzumab (Anti-B7-H3) + and Pembrolizumab (Anti-PD-1)	Increasing eff T-cells	UC	Locally advance M0/M1	AE	Unresectable/Metastatic	Completed (August 2021)
**NCT05645692** (327)	II	240*	PD-1/LAG-3 BsAb (RO7247669) +/- Tiragolumab (Anti-TIGIT)	Increasing eff T cell	Platinum- in eligible UC	Locally advance M0/M1	ORR	Unresectable/Metastatic	Recruiting (Start: April 2023)
**NCT06178601** (329)	II	36*	Disitamab vedotin (anti-HER2 antibody-drug conjugate) + Cadonilimab (PD-1/CTLA-4 BsAb)	HER2-positive tumor apoptosis + Increasing eff T cell	HER2 overexpressing UC	M1	ORR	Metastatic	Recruiting (Start: August 2023)

N, Patient number; M, Metastasis; eff T cell, effector T cell; NMIBC, non-muscle-invasive bladder cancer; BC, Bladder Cancer; TCC, Transitional Cell Carcinoma; UC, Urothelial Carcinoma; BCG, Bacillus Calmette Guerin; CR, Complete Response; CRR, Complete Response Rate; PR, Partial Response; DFS, Disease-Free Survival; DFR, Disease-Free Rate; EFS, Event-Free Survival; Adverse Events; ORR, Objective Response Rate; DLT, Dose-limiting toxicity; *Estimated number.

### Penile cancer

Penile cancer, with over 95% being penile squamous cell carcinoma (PSCC), is a rare malignancy accounting for less than 1% of cancer deaths globally. Although surgery can be effective in the early stages, advanced PSCC often requires systemic treatments like chemotherapy or radiotherapy, which have limited long-term success. New treatments, including targeted IO therapies, are being researched for better outcomes and fewer side effects (19, 343).

Studies of the TME in PSCC using CD3, CD8, and PD-1 markers found that high stromal exhausted cytotoxic T-cells, indicating an “immune excluded” profile, correlated with poorer survival. On the other hand, low stromal cytotoxic CD8+T-cell levels were linked to LN metastasis, and high FOXP3+ Treg-cell levels were associated with lower DFS rates (344–346). Higher CD8+T-cell and FOXP3 Treg levels were also noted in human papilloma virus (HPV)-positive cases, suggesting stronger immune responses and evasion in HPV-associated penile cancer (343). TAMs and TGF-β play crucial roles in promoting angiogenesis and immunotolerance, impacting metastatic progression and treatment resistance in PSCC. Studies in PSCC link high CD68+TAMs densities with better survival and lower regional recurrence risk, whereas high intra-tumoral CD163^+^TAMs correlate with LN metastasis (19, 347).

In various cancers, high TMB and MSI are linked with increased neoantigen expression, which boosts the effectiveness of ICI. However, studies reveal that PSCC typically shows lower TMB and MSI compared to other cancers. Despite this, ICIs may benefit a minority of PSCC cases with TMB greater than 10 mutations per MB, accounting for about 18% of cases. This suggests a potential, albeit limited, role for ICIs in PSCC treatment, especially in select patients with higher TMB levels (99, 348). Similarly, a study showed that PD-L1 expression and MSI status could represent the potential biomarkers in predicting IO efficacy in PSCC (349).

PD-L1 is present in 40-60% of PSCCs, especially in high-risk HPV-negative cases, and is associated with poor outcomes, LN metastasis, and fewer TILs (19). Anti-PD-1 therapy, such as Nivolumab, has shown tumor reduction in HPV-negative case in advanced PSCC, indicating a potential for combining anti-PD-1/PD-L1 agents (350).

ICIs are approved for second-line treatment in metastatic or relapsed penile cancer (351). Trials have shown mixed results; a basket trial with Nivolumab and Ipilimumab involved five penile carcinoma patients with no responses, though two had stable disease, and three progressed (352). Another trial treated three patients with Pembrolizumab, yielding progression in two and a partial response in one with an MSI-H tumor (353). The HERCULES trial demonstrated the effectiveness and safety of first-line ICI with platinum-based chemotherapy in advanced PSCC, identifying HPV16 and TMB as potential biomarkers (354). The ongoing PULSE study is examining avelumab maintenance in metastatic PSCC, with promising initial results (355), and an ongoing phase-II trial is assessing avelumab’s impact on post-platinum therapy progression (356). Additionally, two ongoing studies, including penile carcinoma with other rare cancer types, are underway. The first one evaluates a combination of Nivolumab and Ipilimumab (357), while the second assesses Atezolizumab and Bevacizumab combination therapy (358).

HPV vaccines could be key in preventing and managing PSCC due to HPV’s high prevalence in these cases. While preventive vaccines have reduced cervical cancer rates, their impact on PSCC is unclear. Therapeutic vaccines targeting HPV oncoproteins like E6 and E7 show promise for treating HPV-driven cancers, but studies specific to PSCC are needed (99, 359).

Bintrafusp alfa, a bifunctional fusion protein consisting of the extracellular domain of TGF-β fused to an anti-PD-L1 antibody, has demonstrated clinical activity and manageable safety in HPV-associated cancers. However, the study did not have any participants with PSCC (360). A similar ongoing trial pairs the PRGN-2009 HPV vaccine with Bintrafusp alfa, with early results indicating good tolerability and promising clinical activity against HPV-associated cancers. Grade 3 TRAEs occurred in 9.1% (duodenal hemorrhage), and 18.1% had grade 4 TRAEs (duodenal hemorrhage, pharyngeal mucositis). No treatment-related deaths were reported (361, 362).

ACT, particularly TCR-T and TIL transfer, is gaining traction for HPV-associated cancers. A phase-I trial of TCR-T targeting the HPV-E7 antigen with aldesleukin showed a 50% objective response rate and 41.7% stable disease in metastatic HPV-related cancers. Lower doses had no dose-limiting toxicities, while one occurred at dose level 3, prompting protocol adjustments. Grade 3–4 AEs were primarily from conditioning regimen and aldesleukin. No TCR reactivity against healthy tissues or treatment-related deaths occurred (99, 363). Two ongoing trials are evaluating E7 TCR-T cells in metastatic and locoregionally advanced HPV-associated cancers, including penile cancers (364–366). Another basket phase-I study is assessing a TCR-T therapy targeting MAGE-A1, MAGE-C2, PRAME, and HPV16-E7 antigens presented on special HLA for advanced solid tumors, including PSCC (367). Meanwhile, TILs transfer therapy has shown promise *in vitro* for PSCC, with patient-derived TILs exhibiting anti-tumor activity, though no *in vivo* applications have been reported, yet marking a significant opportunity for future research (99, 368).

HDAC are enzymes critical for epigenetic modifications that regulate T-cell differentiation and function. HDAC inhibitors can enhance antigen presentation, facilitating MHC I-peptide complex formation and boosting PD-L1 expression. A phase-II trial for metastatic SCCs showed an 18% ORR (2/11) and a median PFS of 2.4 months with the combination of vorinostat, an HDAC inhibitor, and Pembrolizumab for PSCC. Pembrolizumab and vorinostat had to be discontinued due to toxicity in 9% and 39% of patients, respectively, with 60% requiring vorinostat dose reduction. Pembrolizumab had expected safety, while vorinostat’s main toxicities were hematologic, gastrointestinal, asthenia, and creatinine increase (369).

Selected IO studies for penile cancer and their targets in TME are shown in **Table 4**.

**Table 4:** Selected IO studies for penile cancer and their targets in the TME.

Study/NCT Number	Phase	N	Therapy	Target in TME	Penile Cancer Type	M status	Primary Endpoint	Therapy Setting	Status
**NCT02834013** (357)	II	818 (total for all rare cancers)	Nivolumab (Anti-PD-1) + Ipilimumab (Anti-CTLA-4)	Increasing eff T cells	Epithelial tumors	M0 or M1	ORR	Metastatic	Active, not recruiting (Start: January 2017)
**HERCULES** **(NCT04224740)** (408)	II	37	Pembrolizumab (Anti-PD-1) + cisplatin/carboplatin+ 5-FU	Increasing eff T cells + directly kill tumor cells and/or hinder their proliferation	Squamous cell carcinoma	Locally advance M0/M1	ORR	Unresectable/Metastatic	Completed (November 2023)
**PEVOsq (NCT04357873)** (369, 409)	II	11	Pembrolizumab (Anti-PD-1) + Vorinostat (HDAC inhibitor)	Increasing eff T cells +enhance antigen presentation/MHC I-peptide complex formation	Squamous cell carcinoma	Recurrent M0/M1	ORR	Metastatic	Active, not recruiting (Start: October 2020)
**NCT03074513** (358)	II	133 (total for all rare cancers)	Atezolizumab (Anti-PD-L1) + Bevacizumab (VEGF inhibitor)	Increasing eff T cells + inhibiting angiogenesis	Squamous cell carcinoma	M1	OR	Metastatic subsequent line	Active, not recruiting (Start: March 2017)
**NCT02858310** (366)	I/II	180* (total for all HPV+ cancers)	E7-TCR-T-Cells	TCR-T cells against E7 protein-positive cancer cells	HPV associated	Locally advance M0/M1	ORR, AE	Metastatic	Recruiting (Star: January 2017)
**NCT04432597** (362)	I/II	39 (total for all HPV+ cancers)	PRGN-2009 HPV vaccine +/- M7824 (Bintrafusp alfa) (Anti-TGFβ/PD-L1 BsAb)	HPV antigen-specific responses+ Increasing eff T cells/tumor growth inhibition	HPV associated	Locally advance M0/M1	Safety, Level increase in tumor infiltrating T-cells	Metastatic	Active, not recruiting (Start: August 2020)

N, Patient number; M, Metastasis; eff T cell, effector T cell; APC, Antigen-presenting cells; RTK; VEGF, Vascular endothelial growth factor; ORR, Objective Response Rate; OR, Objective Response; AE, Adverse Events; HPV, human papillomavirus; VEGF, Vascular endothelial growth factor; PFS, Progression-Free Survival; BsAb, Bispecific antibody; *Estimated number.

### Testicular cancer

Testicular cancer, the most common in men aged 14-44, is increasing in Western countries, with cryptorchidism as the primary risk factor. Germ cell tumors (GCTs) comprise 95% of cases. Surgery and cisplatin-based chemotherapy cure over 90%, but some cases show resistance or relapse (370, 371). IO for refractory testicular GCTs is currently under investigation (18).

Testis is immunologically privileged, protecting germ cells to support spermatogenesis and steroidogenesis. A study noted a “burned-out” testicular tumor, linking spontaneous regression to the immune microenvironment and tumor vascularization. Testicular cancer patients show specific immune responses to cancer/testis antigens via CD8+ and CD4+ T cells, which diminish post-treatment (18, 372).

GCTs exhibit a unique immune cell and cytokine profile, including higher B-cells, DCs, and cytokines compared to normal tissue. TAFs in GCTs secrete factors like VEGF and IL-6 that promote tumor proliferation and metastasis. Also, elevated levels of IFN-α2, IL-2Rα, and IL-16 were linked to poorer survival in GCT patients. Studies have shown that PD-L1 and CTLA-4 are highly expressed in GCTs, though PD-L1 levels do not consistently predict response to IO. Notably, PD-L1 expression on TILs correlates with better prognosis. Mismatch repair deficiency, associated with higher PD-L1, correlates with platinum resistance, highlighting the challenges in improving IO for GCTs (373).

ICI in metastatic GCTs has shown mixed outcomes. A study reported a 33% tumor reduction in a patient treated with anti-PD-1 therapy for embryonal cell carcinoma (374). Other case studies indicate limited success with Nivolumab and Pembrolizumab in platinum-refractory patients, including one partial response with concurrent etoposide (375) and stable disease in metastatic choriocarcinoma treated with Nivolumab. However, rapid disease progression was observed in a patient receiving Pembrolizumab during a phase-II trial (376, 377).

Further clinical trials with Pembrolizumab (378) and combinations of Durvalumab with Tremelimumab (379) in refractory GCTs have not yielded promising results (380). While a study on Avelumab (381) did not meet its primary endpoints in refractory GCTs (382), it demonstrated effectiveness and a favorable safety profile in a separate trial for gestational trophoblastic tumors, suggesting potential for treating chemoresistant cases (18, 383, 384).

Emerging strategies targeting multiple immune checkpoints, such as anti-TIGIT and anti-PD-1 combinations, prompted by studies which found varied expression of these receptors in seminoma samples (385). Another key target is TIM-3, implicated in T-cell exhaustion, suggesting that blocking both PD-1 and TIM-3 could enhance therapy effectiveness where PD-1 inhibition alone fails (386). Additionally, inhibiting LAG-3, which regulates immune response and T-cell activity, in conjunction with PD-1 blockade, has shown promise in various cancers, although some research indicates no significant difference in LAG-3 and TIM-3 expression in testicular GCTs compared to normal tissues (387). Additionally, ICI combinations are being explored (373, 385).

Claudin 6 (CLDN6) has been identified as an ideal target for IO. Its gene, active during fetal development, is silenced in healthy adult tissues but frequently re-expressed in various solid tumors, including GCTs. The BNT211-01 phase-I trial evaluated BNT211, a CLDN6 targeted CAR-T therapy, alone and with a CLDN6 RNA vaccine (CARVac) in patients with CLDN6-positive relapsed/refractory solid tumors, including 13 patients with GCTs. The combination group showed a higher ORR of 57% and DCR of 85%, with durable CAR-T persistence over 100 days. Two patients had dose-limiting toxicities. Most ≥grade 2AEs were from lymphodepletion or liver enzyme elevations (373, 388).

Brentuximab vedotin (BV) is an anti-CD30 antibody conjugate consisting of a chimeric antibody attached to the CD30 cell surface antigen and linked to the cytotoxic antitubulin agent. BV was studied in a phase-II study; seven relapsed/refractory CD30-positive GCT patients received BV. Results included two objective responses and one complete response lasting over four years. The known side effects of BV such as peripheral sensory and motor neuropathy events, were seen (373, 389, 390). Another study with 24 patients showed significant tumor marker reductions, with 11.1% achieving 3-month PFS and 85.7% reaching 6-month OS. However, a case report involving a combination of BV and Pembrolizumab in a heavily pretreated patient led to a complete response but severe toxicities such as grade 3 immune-mediated hepatitis and polyneuropathy (373, 391).

Combination of hypomethylating agents and IO, noting that seminomas’ significant DNA hypomethylation, is linked to high CD8^+^T-cell levels, boosts tumor immunogenicity via increased expression of endogenous retroviruses and IFN-α1 activation (392). Targeting TAMs by inhibiting their recruitment or boosting their anti-tumor functions also holds promise (393).

The selected IO studies for testicular cancer and their targets in the TME are listed in **Table 5**.

**Table 5:** Selected IO studies for testicular cancer and their targets in the TME.

Study/NCT Number	Phase	N	Therapy	Target in TME	Testicular Cancer Type	M status	Primary Endpoint	Therapy Setting	Status
**NCT01461538** (390)	II	24	Brentuximab Vedotin (anti-CD30 antibody conjugate linked to the cytotoxin monomethylauristatin E)	Cell death of CD30-positive cancer cells	CD30-positive GCT	M0/M1	ORR	Adjuvant	Completed (December 2014)
**TROPHIMMUN** **(NCT03135769)** (384)	II	24	Avelumab (Anti-PD-L1)	Increasing eff T cells	Gestational Trophoblastic Neoplasia (Chemo-resistant)	M0/M1	Rate of normalization of hCG	Metastatic	Completed (March 2021)
**BNT211-01 (NCT04503278)** (373, 410)	I	13	BNT211 (CLDN6 targeted CAR-T cell) +/- CARVac (CLDN6 RNA vaccine)	Targeting CLDN6-positive cancer cells via CLDN6-specific CAR-T cells +/- CAR-T cell-amplifying	CLDN6-positive relapsed/refractory GCT	Recurrent/Advanced M0/M1	AE, DLT	Metastatic	Recruiting (Start: September 2020)
**APACHE trial** **(NCT03081923)** (379)	II	36	Durvalumab (Anti-PD-L1) + Tremelimumab (Anti-CTLA-4)	Increasing eff T cells +increased T cell proliferation in LN	GCT (Failure of ≥2 prior chemotherapy regimens)	M1	ORR	Metastatic	Terminated (December 2019)

N, Patient number; M, Metastasis; eff T cell, effector T cell; GCT, Germ cell tumor; SCST, Sex cord-stromal tumors; CBR, Clinical Benefit Rate; ORR, Objective Response Rate; PFS, Progression-Free Survival; CR, Complete Response; DLT, Dose-limiting toxicity.

**Figure 3:**
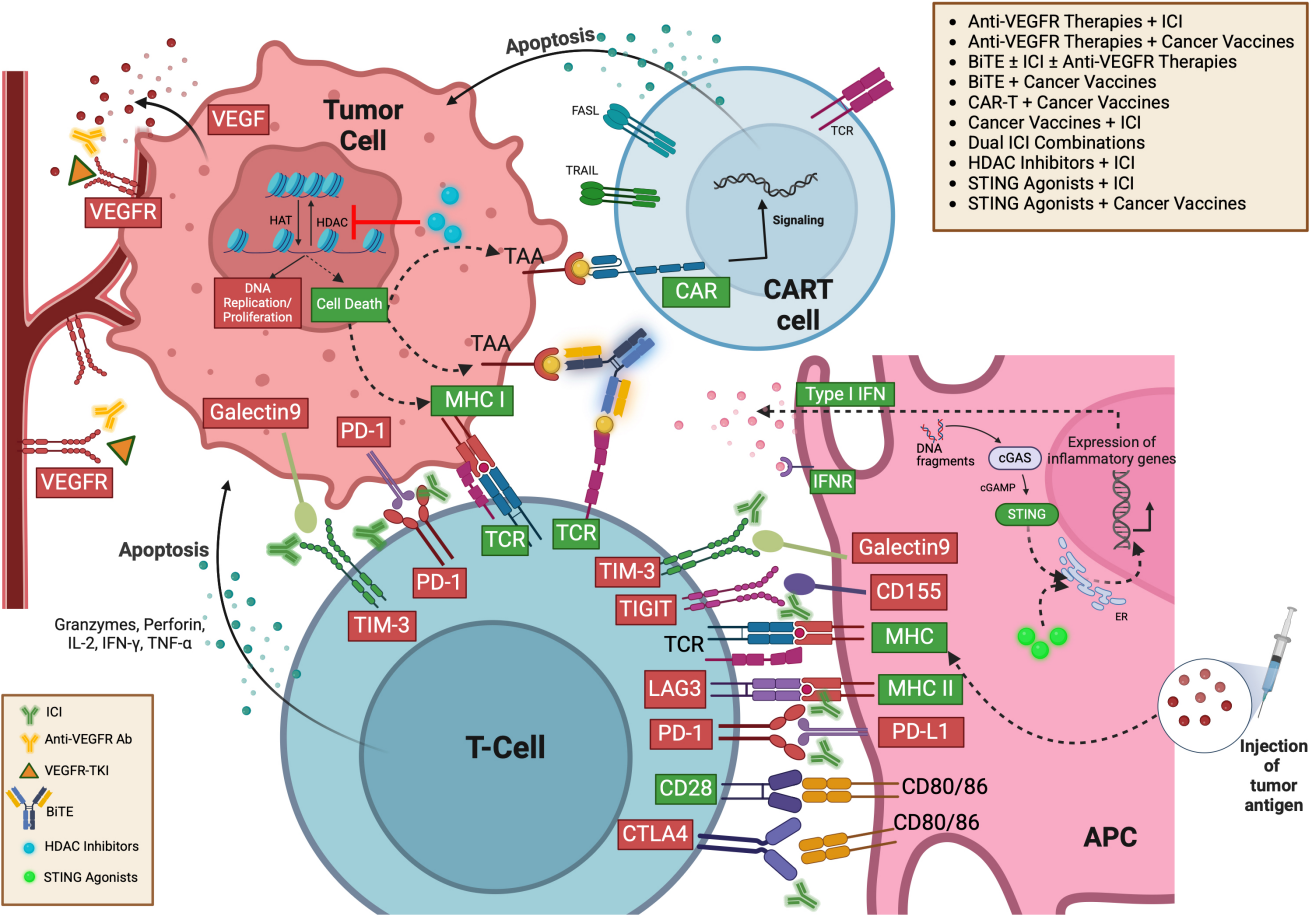
Possible immunotherapy combination strategies targeting TME for GU cancers (413). TME, Tumor microenvironment; GU, Genitourinary; VEGF, Vascular Endothelial Growth Factor; VEGFR, Vascular Endothelial Growth Factor Receptor; HAT, Histone Acetyltransferase; HDAC, Histone Deacetylase; TAA, Tumor-Associated Antigen; MHC I, Major Histocompatibility Complex Class I; PD-1, Programmed Death-1; TCR, T-Cell Receptor; CAR, Chimeric Antigen Receptor; CART, Chimeric Antigen Receptor T-cell; ICI, Immune Checkpoint Inhibitor; BiTE, Bispecific T-cell Engager; STING, Stimulator of Interferon Genes; IFN, Interferon; IFNR, Interferon Receptor; TIM-3, T-cell Immunoglobulin and Mucin-domain containing-3; TIGIT, T cell Immunoreceptor with Ig and ITIM domains; LAG3, Lymphocyte-activation gene 3; PD-L1, Programmed Death-Ligand 1; CD28, Cluster of Differentiation 28; CTLA4, Cytotoxic T-Lymphocyte-Associated Protein 4; MHC II, Major Histocompatibility Complex Class II; CD80/86, Cluster of Differentiation 80/86; APC, Antigen Presenting Cell; cGAS, cyclic GMP-AMP Synthase; cGAMP, Cyclic GMP-AMP; ER, Endoplasmic Reticulum.

IO combinations for GU cancers are schematized in **Figure 3**.

## Conclusion and future directions

IO is revolutionizing the treatment landscape for GU cancers, shifting the focus from conventional approaches to harnessing the immune system’s potential. The intricate crosstalk within the TME plays a pivotal role in shaping cancer progression and therapeutic responses, underscoring the need for a deeper understanding of its cellular and molecular dynamics. As research advances, the integration of novel biomarkers, tailored IO strategies, and optimized combination therapies will be key to overcoming resistance and improving treatment durability. The future of GU cancer therapy lies in refining these approaches to enhance efficacy while minimizing toxicity, ultimately paving the way for more precise, durable, and patient-centered cancer care.
